# TCF3 activates super-enhancer-driven TRIB2 overexpression to suppress ferroptosis and promote hepatoblastoma proliferation

**DOI:** 10.1186/s13046-025-03587-1

**Published:** 2025-12-29

**Authors:** Han Wu, Guoqing Zhu, Qianshu Zhu, Ji Ma, Siwei Mao, Miao Ding, Jiabei Zhu, Xiaochen Tang, Zhixuan Bian, Yuhua Shan, Song Gu, Fenyong Sun, Cizhong Jiang, Qiuhui Pan

**Affiliations:** 1 Department of Clinical Laboratory, Shanghai Children’s Medical Center, School of Medicine, Shanghai Jiao Tong University, Dongfang Road No.1678, Pudong New District, Shanghai, 200127 China; 2 Chongqing Key Laboratory of Human Embryo Engineering and Precision Medicine, NHC Key Laboratory of Birth Defects and Reproductive Health, Center for Reproductive Medicine, Chongqing Health Center for Women and Children, Women and Children’s Hospital of Chongqing Medical University, Chongqing, China; 3 Department of Oncology Surgery, International Diagnosis and Treatment (Special Consultation) Department, Shanghai Children’s Medical Center, School of Medicine, Shanghai Jiao Tong University, Shanghai, China; 4 Department of Clinical Laboratory, Shanghai Tenth People’s Hospital, School of Medicine, Tongji University, Middle Yanchang Road No.301, Jing’an District, Shanghai, 200072 China; 5 Key Laboratory of Spine and Spinal Cord Injury Repair and Regeneration of the Ministry of Education, Orthopaedic Department of Tongji Hospital, School of Life Sciences and Technology, Tongji University, Siping Road No.1239, Yangpu District, Shanghai, 200092 China; 6 Faculty of Medical Laboratory Science, College of Health Science and Technology, Shanghai Jiao Tong University School of Medicine, Shanghai, China; 7 Shanghai Key Laboratory of Clinical Molecular Diagnostics for Pediatrics, Shanghai, China; 8 Hainan Branch, Shanghai Children’s Medical Center, School of Medicine, Shanghai Jiao Tong University, Sanya, Hainan China

**Keywords:** Hi-C, Hepatoblastoma, Epigenetics, Multi-omics, TRIB2, Super-enhancer, KEAP1, NRF2, Reactive oxygen species

## Abstract

**Background:**

Hepatoblastoma (HB) is the most common pediatric liver malignancy with an increasing incidence. However, the functional roles of 3D chromatin organization, epigenetic regulatory factors, and transcriptional reprogramming in HB pathogenesis remain poorly understood.

**Methods:**

​Integrated multi-omics analyses of HB and matched non-tumor tissues were performed, including Hi-C, H3K27ac CUT&Tag, ATAC-seq, and RNA-seq, to construct high-resolution 3D epigenomic maps and identify genes interacting with HB-specific super-enhancers (SEs). Functional assays of identified targets were conducted in cell lines and animal models. The regulatory mechanisms of SEs and upstream transcription factors (TFs) were investigated using CRISPRi-dCas9, 3C-qPCR, ChIP-qPCR, and luciferase reporter assays.

**Results:**

Comprehensive analysis identified TRIB2 as an HB-specific SE-associated oncogene. Functionally, TRIB2 promoted cell proliferation and accelerated tumor growth both in vitro and in vivo. Patients with high TRIB2 expression exhibited advanced PRETEXT stage and metastasis. Mechanistically, TCF3 directly bound to both the TRIB2-SE and its promoter, promoting TRIB2 overexpression. Moreover, TRIB2 conferred resistance to ferroptosis by disrupting KEAP1-mediated ubiquitination of NRF2, thereby stabilizing NRF2 protein and enhancing antioxidant responses. The TCF3-TRIB2-NRF2 axis showed significant co-expression in HB tissues, effectively distinguished HB from normal liver tissues, and was associated with poorer overall survival.

**Conclusions:**

Our findings reveal that TCF3 and SE mediate TRIB2 overexpression to inhibit ferroptosis via the KEAP1-NRF2 pathway and drive HB pathogenesis, providing potential diagnostic and prognostic markers for HB.

**Supplementary Information:**

The online version contains supplementary material available at 10.1186/s13046-025-03587-1.

## Introduction

Hepatoblastoma (HB) is the most common malignant liver tumor in children [1], with an annual incidence growth rate exceeding 4.3% [2]. This embryonal tumor is believed to originate from hepatic progenitor cells [3] and exhibits heterogeneous histological patterns, suggesting a close association with liver development. Notably, HB demonstrates the lowest somatic mutation burden among pediatric cancers (averaging 2.9 mutations per tumor) [4], with most alterations being activating mutations or deletions in *CTNNB1* (encoding β-catenin) [5]. This indicates that mechanisms beyond structural genetic variations may play critical roles in HB pathogenesis [6]. Aberrant epigenetic landscapes may disrupt key signaling pathways that drive tumorigenesis, possibly through changes in chromatin accessibility, the spatial clustering of distal promoters, enhancers, and other *cis*-regulatory elements [7, 8], and alterations in chromatin architecture [9]. However, comprehensive epigenetic data for HB are limited, underscoring the need for further research to provide valuable insights for diagnosis and treatment.

Ferroptosis is an iron-dependent, lipid peroxidation-driven form of programmed cell death with important implications for cancer development, progression, and therapy [10, 11]. Maintaining cellular redox homeostasis is essential for regulating this process [12]. The KEAP1-NRF2 signaling axis serves as a central regulator of the antioxidant response and redox balance [13]. Under basal conditions, KEAP1 targets NRF2 for ubiquitination and proteasomal degradation, limiting its transcriptional activity [14]. Upon oxidative or electrophilic stress, KEAP1 dissociates from NRF2, allowing stabilized NRF2 to translocate into the nucleus and activate cytoprotective genes by binding to antioxidant response elements (ARE) in gene promoters, such as *NQO1* and *HMOX1* [15]. This pathway counteracts lipid peroxidation and confers resistance to ferroptosis, thereby promoting tumor cell survival and chemoresistance in multiple cancers.

Dysregulated gene expression in cancer is often driven by aberrant super-enhancer (SE) activity. SEs are long *cis*-regulatory DNA elements with strong transcriptional activity, composed of clusters of adjacent enhancers [16]. They can mediate transcriptional activation in cancer by recruiting core regulatory transcription factors (TFs) [17]. Genomic regions harboring active SEs are highly enriched with transcription-associated histone marks, such as Histone 3 lysine 27 acetylation (H3K27ac) and Histone 3 lysine 4 monomethylation (H3K4me1), and exhibit elevated chromatin accessibility [17, 18]. Numerous oncogenic TFs exploit SEs to amplify the transcriptional activity of key oncogenes. For instance, KLF7 binds to the *IGF2BP2*-SE to drive *IGF2BP2* overexpression and promote tumor progression in head and neck squamous cell carcinoma (HNSCC) [19], while TP63 and SOX2 co-activate SEs and the promoter of *CCAT1* to drive tumorigenesis in squamous cell carcinomas (SCCs) [20]. Transcription factor 3 (TCF3), a member of the basic helix-loop-helix (bHLH) TF family, is crucial for embryonic development, stem cell maintenance, and carcinogenesis [21]. Nevertheless, the mechanisms by which TCF3 or other key TFs interact with SEs to drive oncogenic transcription remain largely unknown.

Tribbles homolog 2 (TRIB2), an ​​oncogenic​​ pseudokinase, acts as a critical molecular adaptor that recruiting specific E3 ubiquitin ligases to target substrates for degradation, thereby regulating their activity [22]. A notable example​​ is its recruitment of COP1 to promote the proteasomal degradation of C/EBPα [23]. In addition, TRIB2 serves as a scaffold that assembles and regulates components of major signaling pathways, including the PI3K/AKT and MAPK/ERK networks [24, 25]. It has been reported to orchestrate AKT-dependent inactivation of GSK3β, thereby stabilizing β-catenin and promoting chemoresistance in cancer stem cells [26]. ​​Despite the established oncogenic roles of TRIB2 in various malignancies, its upstream regulators, specific functions, and interactions with key oncogenic pathways in HB pathogenesis remain poorly understood and warrant mechanistic investigation.

In this study, integrative multi-omics profiling of HB and matched non-tumor tissues identified TRIB2 as an SE-driven oncogene that promotes HB malignancy both in vitro and in vivo. Mechanistically, TCF3 binds to the TRIB2-SE and its promoter to drive TRIB2 transcriptional activation. Elevated TRIB2 expression confers ferroptosis resistance by maintaining redox homeostasis via the KEAP1-NRF2 pathway, thereby promoting HB tumorigenesis. These findings establish the TCF3-TRIB2-NRF2 axis as a novel regulatory circuit driving HB proliferation and provide a foundation for the development of diagnostic and therapeutic strategies.

## Methods

### HB patients and clinical specimens

Paired HB tissues and adjacent non-tumor liver tissues were collected from HB patients at Shanghai Children’s Medical Center (Shanghai, China) without preoperative chemotherapy or radiotherapy. Complete clinicopathological data were available for all cases. This study was approved by the ethics committee of Shanghai Children’s Medical Center, and written informed consent was obtained from each patient.

### Hi-C library construction

Hi-C libraries were prepared according to previously described methods [27]. (1) Crosslinking: Samples were treated at room temperature with 1% formaldehyde for 10 min, followed by quenching with 0.125 M glycine for 5 min. (2) Lysis: Fixed cells were subsequently disrupted using lysis buffer. (3) Chromatin processing: Endogenous nucleases were inactivated with 0.3% SDS, and chromatin was digested using the MboI restriction enzyme (100 units, New England Biolabs, NEB, Ipswich, MA, USA). (4) Labeling and ligation: DNA ends were labeled with biotin-14-dCTP (Invitrogen, Carlsbad, CA, USA) and ligated with T4 DNA ligase (50 units, NEB). (5) DNA purification: After reversing the crosslinks, ligation products were isolated using the QIAamp DNA Mini Kit (Qiagen, Hilden, Germany). (6) Library preparation: Purified DNA was fragmented by sonication (300–500 bp), subjected to blunt-end repair, A-tailing, and adapter ligation, followed by streptavidin-mediated enrichment of biotinylated fragments and PCR amplification. (7) Sequencing: The final libraries were quality-controlled and sequenced on MGI-Seq platforms (BGI, China).

### Integrated multi-omics analysis

Integrated multi-omics analysis included RNA-seq, Assay for Transposase-Accessible Chromatin using sequencing (ATAC-seq), H3K27ac Cleavage Under Targets and Tagmentation (CUT&Tag), and Hi-C data. RNA-seq was performed on Illumina platforms, and sequencing reads were aligned to GRCh38 using Bowtie2 (v2.5.3) [28]. Differentially expressed genes (DEGs) were identified using DESeq2 (v1.42.1) with significance thresholds of false discovery rate (FDR) < 0.01 and |log fold-change (logFC)| >1. Spearman correlation analysis was performed based on Transcripts Per Million (TPM) values. Public HB datasets (GSE132219 and GSE104766) were analyzed for validation.

For chromatin profiling, ATAC-seq and H3K27ac CUT&Tag data (with two technical replicates) were aligned to the human genome (GRCh38) using Bowtie2 (v2.5.3). Only uniquely mapped reads were used for peak calling with MACS2 (q value < 1e-5). Differentially accessible regions (DARs) calling was performed by DiffBind (v3.12.0) package with FDR < 0.05 and |logFC| >1.5 [29]. Pearson correlation analysis was performed using plotCorrelation from deeptools (v3.5.4).

Hi-C data (with two technical replicates) were processed using HiC-Pro to generate corrected and normalized contact matrices [30]. Chromatin compartments were analyzed with HOMER (v4.11), where genomic regions with positive or negative principal component 1 (PC1) values were classified as A or B compartments, respectively [31]. Topologically associating domains (TADs) calling was identified using Cworld [32], and dynamic TAD boundaries were determined based on Insulation Scores (ISs) [33]. Peakachu (v2.0) was used to detect genome-wide chromatin interaction loops at a 10 kb resolution. Functional regulatory interactions were further screened by predicting enhancer-promoter (E-P) loops using the Activity-by-Contact (ABC) model [34], which integrated chromatin accessibility (ATAC-seq), H3K27ac CUT&Tag, and Hi-C data.

### Cell culture and treatment

HepG2, Huh6, and HEK293T cells were obtained from the Cell Bank of the Type Culture Collection of the Chinese Academy of Sciences (Shanghai, China) and cultured in DMEM (#C11995500BT, Gibco Laboratories) supplemented with 10% fetal bovine serum (FBS, #F0193-500 ml, Sigma, St. Louis, MO, USA) and 1% penicillin-streptomycin solution (#C100C5, NCM Biotech, Suzhou, China) at 37 °C in a 5% CO_2_ incubator. JQ1 was purchased from Solarbio (Beijing, China). Cycloheximide (CHX), ferrostatin-1, Z-VAD-FMK, necrosulfonamide, MG132, RSL3, and Erastin were obtained from MedChemExpress (NJ, USA).

### Lentiviral plasmids and SiRNA sequences

Lentiviral expression plasmids for TRIB2, NRF2, shTRIB2-1, and shTRIB2-2 were purchased from Genomeditech (Shanghai, China). Lentiviral plasmids for TCF3 and shTCF3 were purchased from GeneChem (Shanghai, China). Small interfering RNAs (siRNAs) targeting NRF2 were purchased from GenePharma (Suzhou, China). The sequences used were as follows: shTRIB2-1 (5’-TGGACTCTAGTATGTAAAT-3’), shTRIB2-2 (5’-GCGTTTCTTGTATCGGGAAAT-3’), shTCF3 (5’-GCTGCATGGAGCAGAGGTGAA-3’), and siNRF2 (sense: 5’-GACAGAAGUUGACAAUUAUTT-3’, antisense: 5’-AUAAUUGUCAACUUCUGUCTT-3’).

### Lentiviral transduction

Lentiviral particles were produced by co-transfecting the target plasmid with packaging plasmids (psPAX2 and pMD2G) into HEK293T cells. After 12 h of incubation, the culture medium was replaced, and viral supernatants were collected at 24 and 48 h post-transfection. For infection, HB cells were seeded in 6-well plates and transduced with viral supernatant supplemented with 2 µg/mL polybrene (#H8761, Solarbio). Following 48 h of incubation, stable cell lines were selected using 2 µg/mL puromycin (#HY-B1743A, MedChemExpress).

### Cell proliferation assay

For the CCK-8 assay, a total of 1,000 cells per well were seeded in a 96-well plate. At the indicated time points, the culture medium was replaced with 100 µL of fresh medium containing 10 µL of CCK-8 reagent (#C0043, Beyotime Biotechnology, Shanghai, China) and incubated for 2.5 h at 37 °C. Absorbance at 450 nm was measured using a Synergy2 multimode microplate reader (BioTek Instruments, Winooski, VT, USA).

For the colony formation assay, a total of 1,000 cells per well were plated in a 12-well plate and cultured for 10–12 days. Colonies were washed with phosphate-buffered saline (PBS), fixed with 4% paraformaldehyde, and stained with 0.1% crystal violet. Colony images were captured and quantified using ImageJ software (U.S. National Institutes of Health, Bethesda, MD, USA).

### RNA extraction and real-time quantitative polymerase chain reaction (qRT-PCR) assay

Total RNA was extracted using the Cell/Tissue Total RNA Kit (#M5105, NCM Biotech) according to the manufacturer’s protocol. Reverse transcription was performed using the *Evo M-MLV* RT Mix Kit (#AG11728, Accurate Biology, Hunan, China). All qRT-PCR analyses were conducted using the SYBR Green Premix *Pro Taq* HS qPCR Kit (#AG11718, Accurate Biology). 18S ribosomal RNA (rRNA) served as the internal reference gene. Relative gene expression levels were calculated using the 2^−ΔΔCT^ method. The primer sequences used for qRT-PCR were as follows: TRIB2 (forward: 5′-GACTCCGAACTTGTCGCATTG-3′, reverse: 5′-GGCACGAAAAACGTGGTCT-3′); TCF3 (forward: CCGACTCCTACAGTGGGCTA, reverse: CGCTGACGTGTTCTCCTCG); NFE2L2 (forward: 5′-TCAGCGACGGAAAGAGTATGA-3′, reverse: 5′-CCACTGGTTTCTGACTGGATGT-3′); NQO1 (forward: 5′-GAAGAGCACTGATCGTACTGGC-3′, reverse: 5′-GGATACTGAAAGTTCGCAGGG-3′); HMOX1 (forward: 5′-AAGACTGCGTTCCTGCTCAAC-3′, reverse: 5′-AAAGCCCTACAGCAACTGTCG-3′); 18 S (forward: 5′-CAGCCACCCGAGATTGAGCA-3′, reverse: 5′-TAGTAGCGACGGGCGGTGTG-3′).

### Immunoblotting and Immunoprecipitation (IP)

Cells were lysed on ice for 30 min using RIPA buffer (#P0013B, Beyotime) supplemented with protease inhibitors. After centrifugation at 12,000 rpm for 10 min at 4 °C, protein concentrations in the supernatants were quantified using the BCA Protein Assay Kit (#A55864, Thermo Fisher Scientific, Waltham, MA, USA). Equal amounts of protein were separated by SDS-PAGE and transferred to nitrocellulose membranes (#BS5022001, BioScience, Shanghai, China). Membranes were blocked with 5% bovine serum albumin (BSA) at room temperature for 1 h and then incubated with primary antibodies overnight at 4 °C. The primary antibodies used in this study were anti-TRIB2 (1:1000, #13533S, Cell Signaling Technology, Danvers, MA, USA), anti-TCF3 (1:1000, #67140-1-Ig, Proteintech), anti-NRF2 (1:1000, #T55136S, Abmart, Shanghai, China), anti-KEAP1 (1:500, #TA5266S, Abmart), anti-GAPDH (1:5000, #60004-1-Ig, Proteintech, Wuhan, China), anti-NQO1 (1:1000, #ab28947, Abcam, Cambridge, UK), anti-HO-1 (1:1000, #10701-1-AP, Proteintech), anti-4-Hydroxynonenal (4-HNE, 1:1000, #ab46545, Abcam), anti-BACH1 (1:1000, #ab180853, Abcam), anti-p62 (1:5000, #ab109012, Abcam), anti-FTH (1:1000, #ab314244, Abcam), anti-DMT1 (1:1000, #20507-1-AP, Proteintech), anti-SLC7A11 (1:2000, #12691S, Cell Signaling Technology), anti-TFRC (1:5000, #ab269513, Abcam), anti-GPX4 (1:1000, #67763-1-Ig, Proteintech), anti-ACSL4 (1:5000, #22401-1-AP, Proteintech), anti-α-tubulin (1:5000, #66031-1-Ig, Proteintech), anti-Histone H3 (1:1000, #9715S, Cell Signaling Technology), anti-Ubiquitin (1:1000, #3936S, Cell Signaling Technology), anti-Ubiquitin (linkage-specific K63; 1:1000, #ab179434, Abcam), and anti-Ubiquitin (linkage-specific K48; 1:1000, #ab140601, Abcam). After incubation with secondary antibodies for 1 h, chemiluminescent signals were detected and recorded accordingly.

For IP assays targeting TRIB2 (1:200, #13533S, Cell Signaling Technology) and NRF2 (1:200, #16396-1-AP, Proteintech), cells were lysed on ice for 30 min using cell lysis buffer (#P0013B, Beyotime Biotechnology, Shanghai, China) supplemented with protease inhibitors. After centrifugation, the lysates were incubated with the corresponding antibody and protein A/G magnetic beads (#HY-K020, MedChemExpress) overnight at 4 °C. The beads were then washed five times with cell lysis buffer, and bound proteins were analyzed by immunoblotting.

### Nuclear and cytoplasmic extraction

Nuclear and cytoplasmic proteins were isolated using the Nuclear Protein Extraction Kit (#R0050, Solarbio) according to the manufacturer’s protocol. α-tubulin and Histone H3 were used as cytoplasmic and nuclear markers, respectively.

### Immunohistochemistry (IHC)

Immunohistochemical staining was performed on paraffin-embedded sections from HB tissues and paired non-tumor tissues. Sections were deparaffinized in xylene and rehydrated through graded ethanol solutions. Endogenous peroxidase activity was blocked with 3% methanol-H_2_O_2_. Antigen retrieval was ​then​ performed using 10 mmol/L sodium citrate solution. The sections were then incubated with primary antibodies at 4 °C overnight, followed by incubation with species-matched secondary antibodies at 37 °C for 1 h. Immunoreactivity was visualized using a diaminobenzidine chromogenic substrate, followed by ​hematoxylin.

### 3C-qPCR assay

Chromatin conformation in paired HB and adjacent non-tumor liver tissue was analyzed using the 3C-qPCR method [35]. After formaldehyde crosslinking, genomic DNA was digested with Csp6I, AseI, or AlwNI (NEB). Primer pairs were designed upstream of the enzyme restriction sites, and qPCR was used for quantification. GAPDH internal primers were used as negative controls and normalization references. The primer sequences are listed in Supplementary Table 1.

### CRISPR interference (CRISPRi)

Short guide RNA (sgRNA)​​ sequences targeting the predicted TRIB2 enhancer elements were designed using ​CRISPOR (https://crispor.gi.ucsc.edu/crispor.py) ​​ [36] and cloned into the ​CMV-dCas9-KRAB-SV40 vector (GeneChem). HB cells​ were transfected with the constructed plasmids using​ Lipofectamine 2000 (#11668019, Invitrogen). TRIB2 expression levels were quantified by qRT-PCR 48 h after transfection​. The sgRNA sequences are listed in Supplementary Table 2.

### Chromatin immunoprecipitation-qPCR (ChIP-qPCR) assay

ChIP assays were performed using the SimpleChIP Plus Kit (#56383S, Cell Signaling Technology) with 4 × 10^6^ cells per reaction. Cells were crosslinked with 1% formaldehyde for 10 min and neutralized with glycine for 5 min. After resuspension, chromatin was sonicated into appropriate fragment sizes and immunoprecipitated overnight at 4 °C with specific antibodies against TCF3 (#12258S, Cell Signaling Technology), bromodomain-containing protein 4 (BRD4, #13440S, Cell Signaling Technology), RNA polymerase II (RNA Pol II, #ab5408, Abcam), or H3K27ac (#ab4729, Abcam). The precipitated DNA was eluted, purified, and quantified by qPCR. Primer sequences are provided in Supplementary Table 3.

### Luciferase reporter assay

To investigate the regulatory effect of TCF3 on the TRIB2 promoter, the wild-type (WT) promoter fragment containing putative TCF3-binding sites and a corresponding mutant (MUT) fragment were cloned into the pGL3-basic vector. HEK293T cells at 60% confluence in 12-well plates were co-transfected with the indicated luciferase reporter plasmids and TCF3 regulators using Lipofectamine 2000. After 48 h, luciferase activity was measured using the Bio-Lumi Firefly Luciferase Assay Kit (#RG042S, Beyotime). Relative luciferase activity was normalized to cell viability, which was determined using the CellTiter-Lumi Plus Luminescent Cell Viability Assay Kit (#C0068S, Beyotime).

### Lipid reactive oxygen species (ROS) assay

Cells were trypsinized, washed with PBS, and resuspended in 500 µL PBS containing 1 µM BODIPY-C11 (#HY-D1301, MedChemExpress). After incubation for 30 min at 37 °C in the dark, lipid ROS levels were analyzed using a CytoFLEX flow cytometer (Beckman Coulter, California, USA) and quantified with FlowJo v10.8.1 Software (BD Life Sciences).

### Immunofluorescence

Cells seeded on confocal dishes (5,000 cells per dish) were fixed with 4% paraformaldehyde (#P0099, Beyotime) for 15 min, permeabilized with Triton X-100 (#ST1723, Beyotime) for 10 min, and blocked with QuickBlock buffer (#P0260, Beyotime) for 10 min. Primary antibodies were applied and incubated overnight at 4 °C. After PBS washes, cells were incubated with fluorophore-conjugated secondary antibodies in the dark for 30 min. Nuclei were counterstained with DAPI (#C1005, Beyotime) for 5 min. Images were captured using a Leica STELLARIS microscope.

### Xenograft tumor assay

Male nude mice (4 weeks old) were subcutaneously injected with 1 × 10^7^ cells (*n* = 6 per group). After 28 days, the mice were euthanized by cervical dislocation, and tumors were excised and weighed. Tumor volumes were calculated using the formula 0.5 × length × width^2^. All animal procedures were approved by the Shanghai Children’s Medical Center and were conducted in accordance with the Guidelines for the Care and Use of Animals for Scientific Research.

### Propidium Iodide (PI) staining​

Cells cultured in 24-well plates were stained with PI (#G1021, Servicebio, Wuhan, China) in the dark for 10 min. Stained cells were observed and imaged using a Leica fluorescence microscope.

### Malondialdehyde (MDA), Glutathione (GSH), and Labile Iron Pool (LIP) assay

The levels of MDA, GSH, and the LIP were measured using commercial assay kits: MDA with the Lipid Peroxidation MDA Assay Kit (#S0131S, Beyotime), GSH with the GSH and GSSG Assay Kit (#S0053, Beyotime), and LIP with the Cellular Red Fluorescent Ferrous Ion Assay Kit with RhoNox-6 (#S1070S, Beyotime).

### Statistical analysis

Statistical analyses were performed using GraphPad Prism 9.5.1 (GraphPad Software, San Diego, CA, USA). Data from at least three independent experiments are presented as mean ± standard deviation (SD). Differences between two groups were analyzed using independent or paired t-tests, while comparisons among three or more groups were performed using one-way ANOVA. Survival analysis of clinical specimens was conducted using the Kaplan-Meier method. Statistical significance was defined as *p < 0.05, **p < 0.01, ***p < 0.001, or ****p < 0.0001.

## Results

### Global 3D epigenome reprogramming in HB pathogenesis

To comprehensively characterize the epigenetic landscape and chromatin architectural alterations during HB development, we performed ATAC-seq, H3K27ac CUT&Tag, Hi-C (each with two technical replicates), and RNA-seq (with five biological replicates) analyses of HB and paired adjacent non-tumor tissues. The clinical characteristics of specimens from HB patients are provided in Supplementary Table 4. All replicates demonstrated high reproducibility (Supplementary Fig. 1A-C). Hi-C analysis generated 956 million and 857 million valid chromatin interaction pairs for HB and non-tumor tissue, respectively (Supplementary Fig. 1D). Analysis of interaction patterns revealed a significantly higher interchromosomal contact fraction in HB (cis/trans ratio: 0.885 vs. 0.993 in non-tumor tissue; Supplementary Fig. 1D), indicating stronger interchromosomal interactions in HB.

**Fig. 1:**
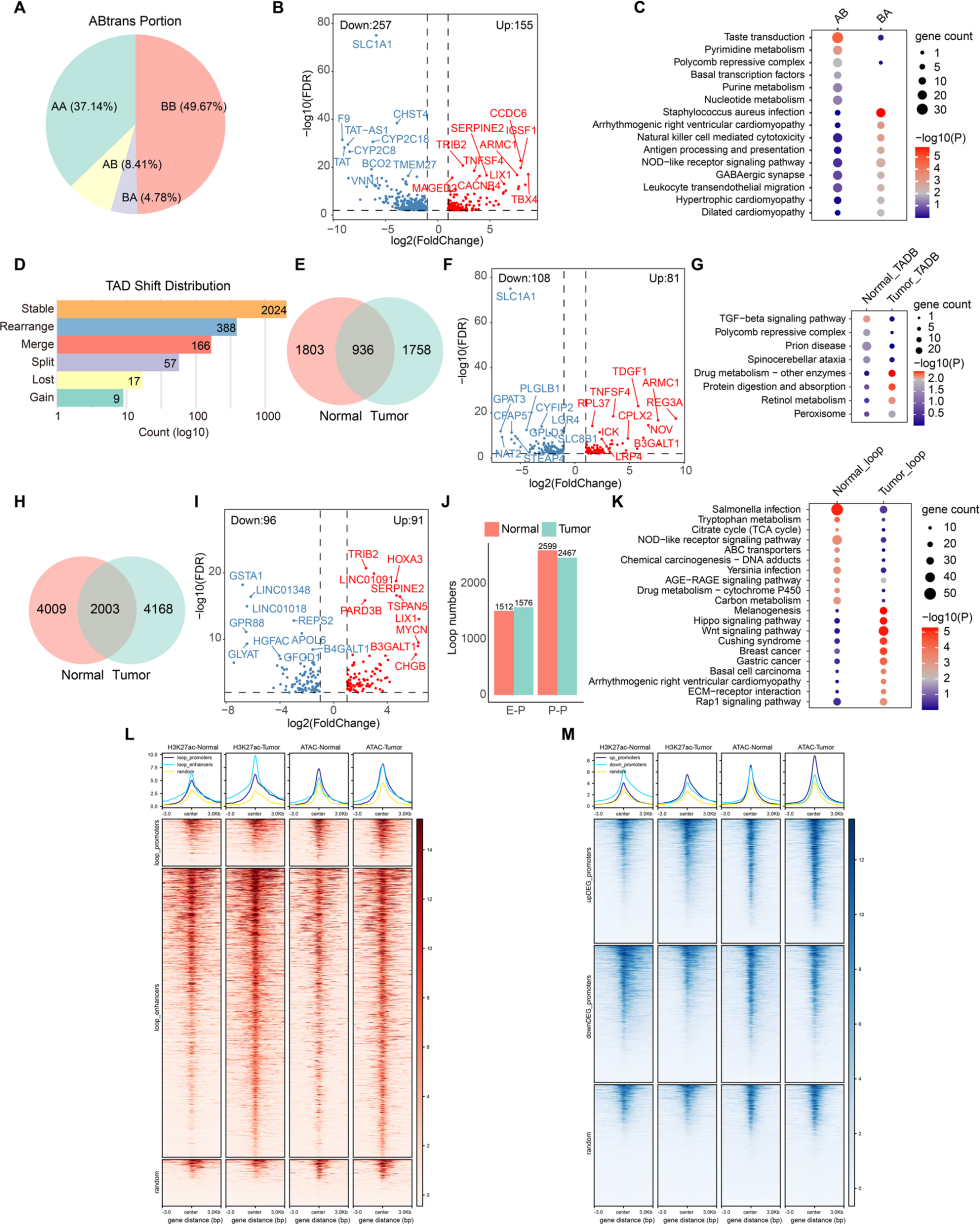
3D genome reorganization in HB tumorigenesis. ​**A** Proportions of A/B compartment switching types. **B** Volcano plot of DEGs associated with compartment switching. **C**​​ KEGG enrichment of genes in switched compartments. **D**​ Distribution of TAD structural variations. **E**​​ Venn diagram of tissue-specific TAD boundaries. **F**​ Volcano plot of DEGs associated with tissue-specific TAD boundaries. **G**​ KEGG enrichment of genes flanking tissue-specific TAD boundaries. **H** Venn diagram of tissue-specific chromatin loops. **I**​​ Volcano plot of DEGs associated with tissue-specific loops. **J** Quantification of chromatin loop subtypes. E-P: enhancer-promoter; P-P: promoter-promoter. **K**​ KEGG enrichment of target genes regulated by tissue-specific E-P loops. **L**-**M** H3K27ac and ATAC-seq heatmaps at HB-specific E-P loop anchors (**L**)​​ and promoters of DEGs (**M**)​​

At the megabase scale, chromatin compartment analysis demonstrated spatial segregation of active (Compartment A) and repressive (Compartment B) genomic domains. Compartment A correlates with open chromatin and transcriptional activity, whereas Compartment B is associated with gene repression and heterochromatic features [37]. During HB tumorigenesis, 13.19% of compartments underwent switching, including A-to-B transitions (8.41%) and B-to-A transitions (4.78%) (Fig. 1A). These dynamic transitions occurred genome-wide and were particularly enriched on chromosomes 2–6, 9, 10, 16, and 17 (Supplementary Fig. 1E). Compartment-switched genes exhibited distinct transcriptional biases, whereby A-to-B transitions were associated with pronounced transcriptional silencing (65% downregulation; Supplementary Fig. 1F). We identified 412 compartment switch-associated DEGs, including 155 upregulated and 257 downregulated genes, such as TRIB2, SERPINE2, and SLC1A1 (Fig. 1B). Kyoto Encyclopedia of Genes and Genomes (KEGG) pathway analysis showed that A-to-B genes were mainly involved in Polycomb repressive complex 2 (PRC2) regulation, while B-to-A genes were enriched in immune surveillance pathways, including natural killer cell mediated cytotoxicity and antigen processing and presentation (Fig. 1C). 

TADs are conserved units of chromosomal organization that spatially constrain regulatory interactions to ensure precise gene expression control [38]. During HB progression, most TAD structures remained conserved (*n* = 2024). Among dynamic TADs, rearrangement (*n* = 388) emerged as the predominant alterations, suggesting local chromatin loop restructuring (Fig. 1D). During malignant transformation, aberrant TAD boundaries can promote reconfigured E-P interactions that dysregulate transcriptional programs [39]. We identified 1,758 HB-specific and 1,803 non-tumor-specific TAD boundaries, containing 81 and 108 DEGs, respectively, such as TDGF1, TNFSF4, and REG3A (Fig. 1E, F). HB-specific TAD boundaries displayed a higher proportion of transcriptional activation compared with non-tumor-specific boundaries (Supplementary Fig. 1G), consistent with epigenetic derepression at oncogenic loci. KEGG enrichment analysis revealed that non-tumor-specific boundary genes were associated with TGF-β signaling and PRC2 functions, whereas HB-specific boundary genes were involved in metabolic reprogramming pathways, including retinol metabolism, drug metabolism, and peroxisome-related pathways associated with lipid metabolism and ROS homeostasis (Fig. 1G).

High-resolution Hi-C maps revealed chromatin loops that typically connect E-P pairs, and dysregulation of these interactions can promote tumorigenesis [27, 40]. Chromatin loop analysis identified 4,168 HB-specific and 4,009 non-tumor-specific chromatin interactions (Fig. 1H), anchoring 187 DEGs, including TRIB2, SERPINE2, and MYCN (Fig. 1I). Compared with adjacent non-tumor tissue, HB samples exhibited a higher abundance of E-P chromatin loops (Fig. 1J). HB-specific E-P interactions targeted core oncogenic pathways such as Hippo and Wnt signaling, as well as solid tumor-related pathways (breast, basal cell, and gastric cancer), extracellular matrix (ECM)-receptor interaction, and focal adhesion (Fig. 1K). Notably, HB-specific regulatory anchors showed coordinated epigenetic activation, characterized by concurrent increases in H3K27ac enrichment and chromatin accessibility at both promoters and enhancers (Fig. 1L; Supplementary Fig. 1H, I). Similar changes were observed at promoters of transcriptionally upregulated genes in HB, where enhanced H3K27ac enrichment and open chromatin established a permissive transcriptional environment (Fig. 1M; Supplementary Fig. 1J, K). Collectively, these results demonstrate that genome-wide 3D chromatin reorganization, including compartment switching, TAD boundary restructuring, and loop rewiring, creates a permissive chromatin landscape that facilitates coordinated epigenetic activation and transcriptional reprogramming of key oncogenic pathways, thereby driving HB pathogenesis.​.

### Integrated multi-omics screening identified TRIB2 as an SE-driven oncogene

**Fig. 2:**
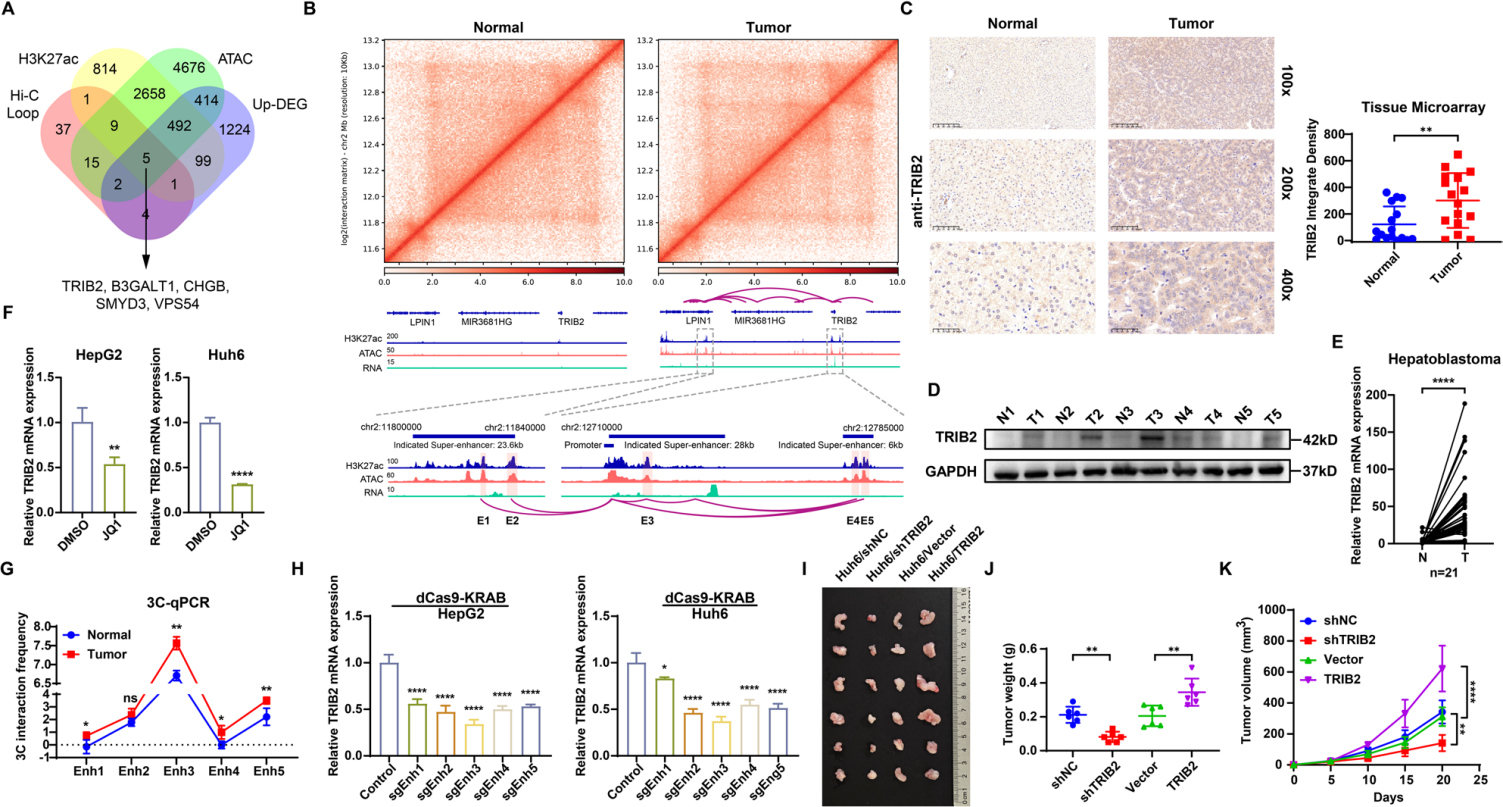
SE-P looping drives TRIB2 oncogene activation in HB. **A** Venn diagram of 5 genes concurrently regulated through 3D chromatin reconfiguration, epigenetic activation, and transcriptional upregulation in HB. **B** Hi-C interaction map of 5 tumor-specific E-P loops anchored at the TRIB2 promoter. **C**-**E** Validation of TRIB2 high expression in HB clinical specimens via IHC staining (n = 16 paired samples; ​**C**), Western blot (n = 5 paired samples; ​**D**), and qRT-qPCR (n = 21 paired samples; ​**E**). **F** TRIB2​​ transcriptional suppression by JQ1 (10µM, 24 h) in HB cells (n = 3 independent biological replicates). **G** 3C-qPCR analysis of the interaction between the candidate enhancers and TRIB2 promoters (n = 3 independent biological replicates). **H** TRIB2 expression​ following enhancer-targeting by CRISPRi-dCas9 (n = 3 independent biological replicates). **I** Tumors dissected from nude mice (n = 6 mice per group) injected with Huh6 cells with or without TRIB2 knockdown/overexpression. **J** Primary tumor weights at the end of the experiment. **K** Tumor growth curves based on tumor volumes measured on the indicated days

To identify oncogenic drivers directly influenced by HB-specific chromatin remodeling, we integrated HB-specific H3K27ac peaks, ATAC-seq peaks, Hi-C loops, and transcriptomic upregulation profiles. After intersecting these datasets, we identified five key genes that simultaneously regulated through 3D chromatin reconfiguration, epigenetic activation, and transcriptional upregulation. These genes included TRIB2, VPS54, SMYD3, CHGB, and B3GALT1 (Fig. 2A). Among them, TRIB2 exhibited the highest expression level in HB and showed the most significant overexpression difference compared with non-tumor tissues (Supplementary Fig. 2A), establishing it as the top-priority oncogenic driver candidate for further investigation.​.

High-resolution Hi-C chromatin interaction analysis revealed five enhancer elements located within SE domains that formed HB-specific loops with the TRIB2 promoter (Fig. 2B). ​This spatial configuration provides a plausible mechanism for the marked upregulation of TRIB2 in tumors. The finding was supported by consistent overexpression evidence from IHC (Fig. 2C, Supplementary Fig. 2B), western blotting (Fig. 2D), and qRT-PCR (Fig. 2E) analyses in clinical HB specimens. Considering the established role of BRD4, a BET family protein, in anchoring SEs to drive oncogene transcription [41], pharmacological inhibition using the BET inhibitor JQ1 significantly reduced TRIB2 expression in HB cells (HepG2 and Huh6; Fig. 2F). Additionally, ChIP-qPCR analysis showed decreased occupancy of BRD4 and RNA Pol II at enhancer elements after JQ1 treatment (Supplementary Fig. 2C, D), confirming the essential role of the TRIB2-SE in transcriptional regulation. 3C-qPCR analysis detected enhanced interactions between the TRIB2 promoter and enhancer elements in HB compared with non-tumor tissue (Fig. 2G). Direct functional inhibition using CRISPRi-dCas9-mediated repression of these enhancer elements significantly reduced TRIB2 expression, supporting their regulatory function (Fig. 2H). ​Functional validation assays, including CCK-8 viability (Supplementary Fig. 2E, G), colony formation (Supplementary Fig. 2F, H), and subcutaneous xenograft experiments (Fig. 2I-K), demonstrated that TRIB2 knockdown suppressed proliferation both in vitro and in vivo, whereas TRIB2 overexpression conversely enhanced proliferative capacity. Collectively, these findings indicate that TRIB2-SE exerts a positive regulatory effect on TRIB2 transcriptional activation.

### TRIB2 depletion inhibits HB proliferation by inducing ferroptosis

**Fig. 3:**
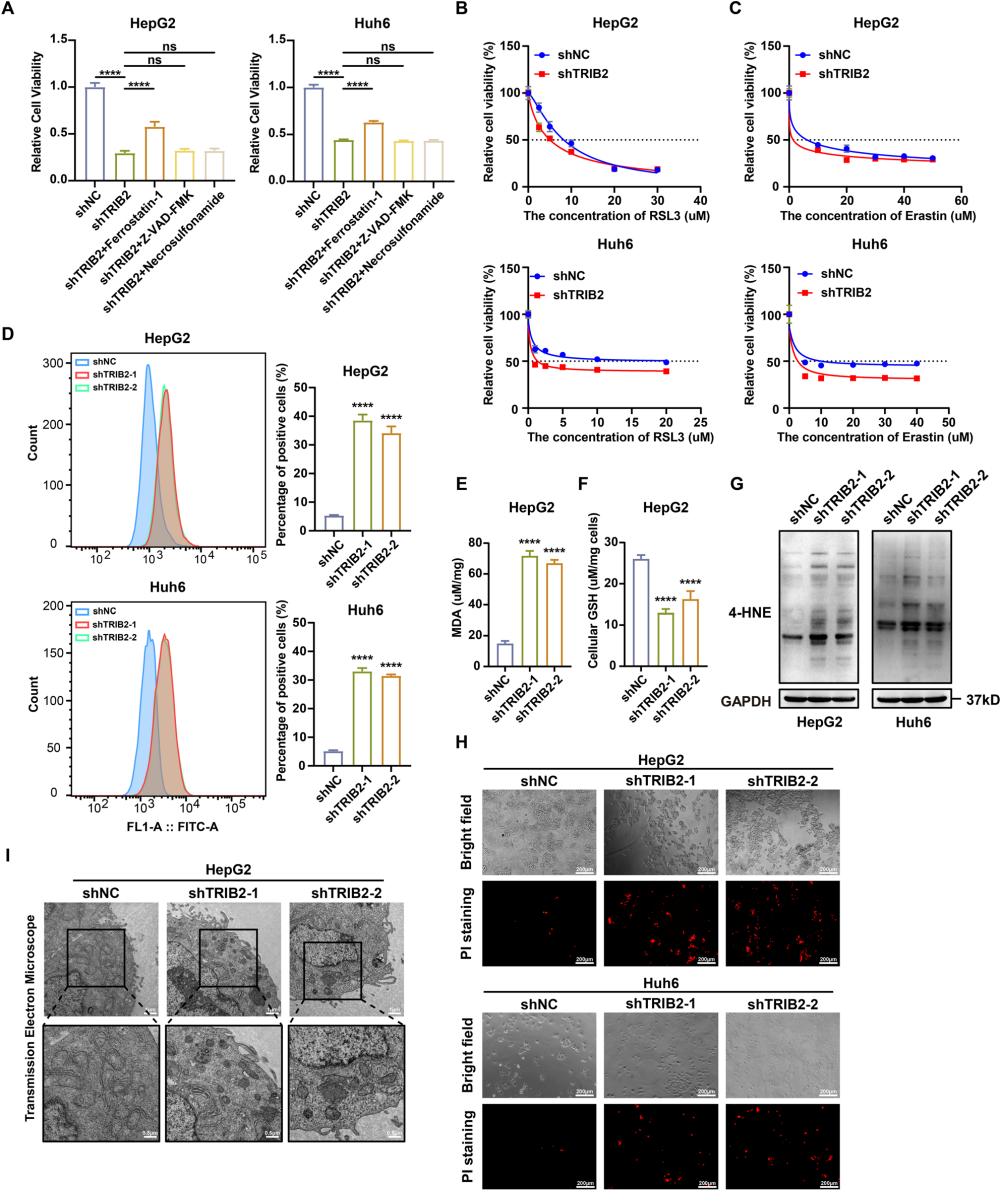
Inhibition of TRIB2 promotes ferroptosis in HB cells. **A**​​ Viability of HB cells with or without TRIB2 knockdown treated with ferroptosis inhibitor ferrostatin-1 (2 µM), apoptosis inhibitor Z-VAD-FMK (10 µM), or necroptosis inhibitor necrosulfonamide (0.5 µM) (n = 5 independent biological replicates). **B**-**C**​​ Dose-response curves for HB cells with or without TRIB2 knockdown treated with RSL3 (**B**) or Erastin (**C**) (n = 5 independent biological replicates). **D**​​ Flow cytometry quantification of lipid ROS using BODIPY-C11 probe oxidation in HB cells with or without TRIB2 knockdown (n = 3 independent biological replicates). **E**-**F**​​ MDA (**E**) or GSH (**F**) levels in HepG2 cell lysates with or without TRIB2 knockdown (n = 3 independent biological replicates). **G** Western blots of 4-HNE protein expression in HB cells with or without TRIB2 knockdown. **H** Cell death measured by PI staining of the HB cells with or without TRIB2 knockdown, scale bar, 200 μm. **I** Transmission electron micrographs showing mitochondrial ultrastructure in HepG2 cells with or without TRIB2 knockdown, scale bar, upper 1 μm, lower ‌500 nm

To investigate the functional role of TRIB2 in regulating HB cell death, we treated TRIB2-knockdown HB cells with the ferroptosis inhibitor ferrostatin-1, the apoptosis inhibitor Z-VAD-FMK, or the necroptosis inhibitor necrosulfonamide. Only ferrostatin-1 markedly rescued cell viability, while the other inhibitors provided minimal protection (Fig. 3A, Supplementary Fig. 3A), indicating that TRIB2 depletion sensitizes HB cells to ferroptosis. Consistently, TRIB2-knockdown HB cells displayed increased sensitivity to the ferroptosis inducers RSL3 and Erastin, as evidenced by decreased IC50 values in dose-response assays (Fig. 3B, C). Ferroptotic lipid peroxidation was elevated in TRIB2-knockdown cells, as shown by increased oxidation of the BODIPY-C11 probe (Fig. 3D) and accumulation of lipid peroxidation end-products, including MDA (Fig. 3E, Supplementary Fig. 3B) and 4-HNE protein adducts (Fig. 3G). Concurrently, reduced GSH levels reflected a compromised antioxidant capacity (Fig. 3F, Supplementary Fig. 3C), indicating disruption of redox homeostasis. Measurement of the LIP further revealed that TRIB2 knockdown significantly increased intracellular iron levels, suggesting that TRIB2 depletion enhances ferroptosis susceptibility by elevating iron availability (Supplementary Fig. 3D). PI staining confirmed increased cell death (Fig. 3H, Supplementary Fig. 3E), and transmission electron microscopy revealed typical ferroptotic ultrastructural features, including mitochondrial matrix condensation and cristae disintegration (Fig. 3I, Supplementary Fig. 3F). Together, these findings establish TRIB2 as a ferroptosis suppressor that protects HB cells by inhibiting lipid peroxidation.

### TRIB2 activates the KEAP1-NRF2 pathway via competitive disruption of NRF2 ubiquitination

**Fig. 4:**
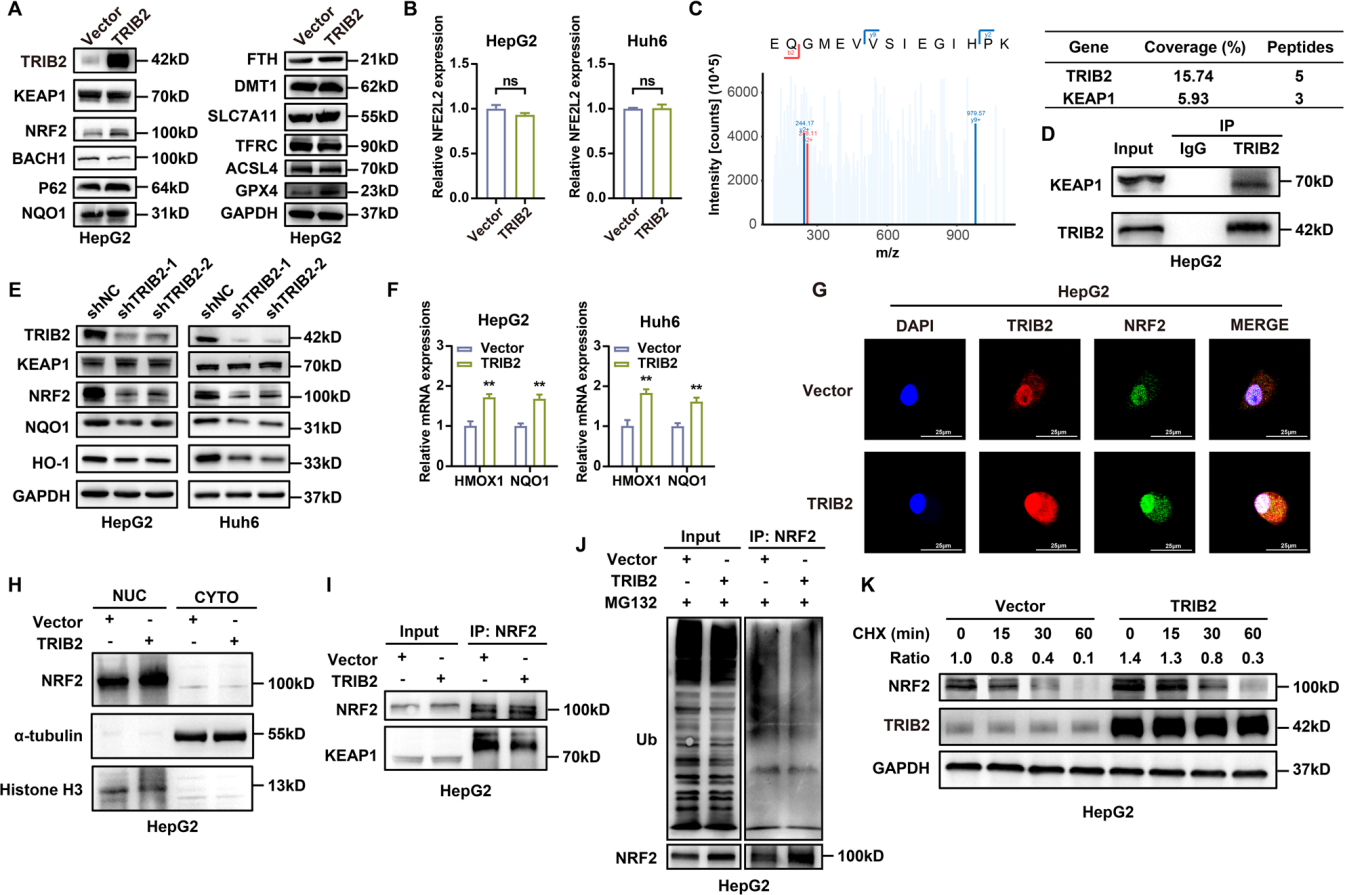
TRIB2 stabilizes NRF2 through competitive disruption of KEAP1-mediated ubiquitination. **A**​​ Western blots of ferroptosis-related regulators in HepG2 cells with or without TRIB2 overexpression. **B** qRT-PCR analysis of NFE2L2 in HB cells with or without TRIB2 overexpression (n = 3 independent biological replicates). **C** Mass spectrometry identification of KEAP1 peptides in TRIB2 immunoprecipitates from HepG2 cells. **D** IP of TRIB2 to verify the interaction between TRIB2 and KEAP1 in HepG2 cells. **E** Western blots of NRF2, HO-1, and NQO1 in HB cells with or without TRIB2 knockdown. **F** qRT-PCR analysis of HMOX1 and NQO1 in HB cells with or without TRIB2 overexpression (n = 3 independent biological replicates). **G** Immunofluorescence imaging of NRF2 in HepG2 cells with or without TRIB2 overexpression (cyan: NRF2; red: TRIB2; blue: DAPI). scale bar, 25 μm. **H** Western blots of the HepG2 cell lysates by nuclear-cytoplasmic fractionation with or without TRIB2 overexpression. Markers: Histone H3 (nucleus), α-tubulin (cytoplasm). **I** IP of NRF2 to verify the interaction between NRF2 and KEAP1 in HepG2 cells with or without TRIB2 overexpression. **J** IP of NRF2 to verify NRF2 ubiquitination under MG132 treatment (10 µM, 6 h) in HepG2 cells with or without TRIB2 overexpression. **K** Western blots of the decay rate of NRF2 protein detected at the indicated time points under the treatment of CHX (100 µg/ml) in HepG2 cells with or without TRIB2 overexpression

To explore the downstream mechanism through which TRIB2 regulates ferroptosis, we screened key effectors involved in iron metabolism (TFRC, DMT1, FTH), redox homeostasis (KEAP1, NRF2, BACH1, NQO1, HO-1), and ferroptosis execution (SLC7A11, GPX4, ACSL4; Fig. 4A, Supplementary Fig. 4A). Notably, TRIB2 overexpression in HB cells markedly increased NRF2 protein levels, whereas its mRNA remained unchanged according to qRT-PCR analysis (Fig. 4B), suggesting post-translational regulation. IP coupled with mass spectrometry in HB cells (Fig. 4C) revealed a direct physical interaction between TRIB2 and KEAP1, which was further validated by western blotting (Fig. 4D, Supplementary Fig. 4B, C). These findings suggested that TRIB2 competitively disrupts the KEAP1-NRF2 interaction, thereby reducing KEAP1-mediated NRF2 ubiquitination and stabilizing NRF2 protein.

Consistent with this mechanism, TRIB2 knockdown reduced NRF2 protein levels and downregulated its downstream targets HMOX1 (encoding HO-1 protein) and NQO1 at both mRNA and protein levels (Fig. 4E, F). Immunofluorescence and nuclear-cytoplasmic fractionation assays in HB cells showed that TRIB2 overexpression promoted NRF2 nuclear translocation (Fig. 4G, H, Supplementary Fig. 4D, E). Mechanistically, TRIB2 overexpression suppressed the formation of the KEAP1-NRF2 complex (Fig. 4I, Supplementary Fig. 4F) and decreased NRF2 ubiquitination (Fig. 4J, Supplementary Fig. 4G). To precisely determine which ubiquitin linkage type was affected by TRIB2, ubiquitination assays using linkage-specific antibodies (K48 and K63) were performed. The results showed that TRIB2 overexpression primarily reduced K48-linked ubiquitination of NRF2 (Supplementary Fig. 4H), while K63-linked ubiquitination showed minor changes (Supplementary Fig. 4I). Consistent with the known function of KEAP1 in K48-linked polyubiquitination of NRF2 [42, 43], this result further validates our finding that TRIB2 impairs this process by competitively binding to KEAP1 and disrupting the KEAP1-NRF2 interaction. Moreover, the CHX chase assay demonstrated that TRIB2 prolonged the half-life of NRF2 protein (Fig. 4K, Supplementary Fig. 4J), confirming that the TRIB2-KEAP1 interaction suppresses NRF2 degradation. Collectively, these findings indicate that TRIB2 stabilizes NRF2 by competitively disrupting KEAP1-mediated ubiquitination, thereby activating the NRF2 antioxidant pathway and suppressing ferroptosis in HB.

### NRF2 reverses TRIB2 deficiency-induced ferroptosis and proliferation defects

**Fig. 5:**
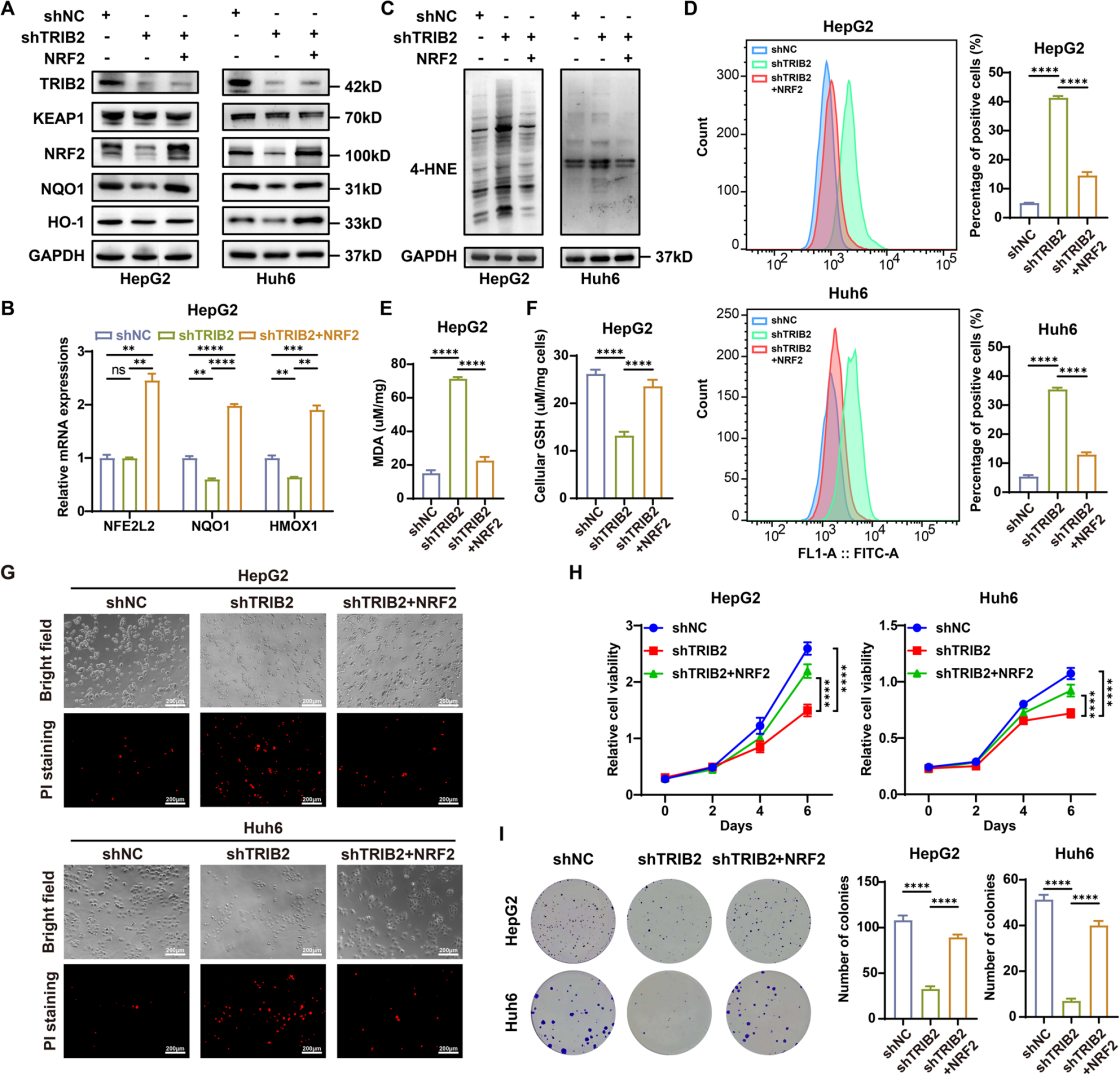
NRF2 reconstitution reverses ferroptosis and proliferative defects caused by TRIB2 depletion. **A** Western blots of NRF2, HO-1, and NQO1 in TRIB2-knockdown HB cells with or without NRF2 overexpression. **B** qRT-PCR analysis of NFE2L2, HMOX1, and NQO1 in TRIB2-knockdown HepG2 cells with or without NRF2 overexpression (n = 3 independent biological replicates). **C** Western blots of 4-HNE protein expression in TRIB2-knockdown HB cells with or without NRF2 overexpression. **D** Flow cytometry quantification of lipid ROS using BODIPY-C11 probe oxidation in TRIB2-knockdown HB cells with or without NRF2 overexpression (n = 3 independent biological replicates). **E**-**F** MDA (**E**) or GSH (**F**) levels in TRIB2-knockdown HepG2 cell lysates with or without NRF2 overexpression (n = 3 independent biological replicates). **G** Cell death measured by PI staining of the TRIB2-knockdown HB cells with or without NRF2 overexpression, scale bar, 200 μm. **H** CCK-8 assay in TRIB2-knockdown HB cells with or without NRF2 overexpression at the indicated time points (n = 5 independent biological replicates). **I** Colony formation assay and quantification in TRIB2-knockdown HB cells with or without NRF2 overexpression (n = 3 independent biological replicates)

To functionally validate NRF2 as the key downstream mediator of TRIB2, we simultaneously knocked down endogenous TRIB2 and overexpressed exogenous NRF2 in HB cells. Ectopic NRF2 expression completely restored HO-1 and NQO1 levels, which had been downregulated by TRIB2 depletion, at both mRNA and protein levels (Fig. 5A, B, Supplementary Fig. 5A), confirming NRF2-dependent transcriptional reactivation. Exogenous NRF2 also reversed lipid peroxidation induced by TRIB2 knockdown, as shown by reduced oxidation of the BODIPY-C11 probe (Fig. 5D) and decreased accumulation of lipid peroxidation end-products, including MDA (Fig. 5E, Supplementary Fig. 5C) and 4-HNE protein adducts (Fig. 5C, Supplementary Fig. 5B). Concurrently, increased GSH levels indicated restored antioxidant capacity (Fig. 5F, Supplementary Fig. 5D), suggesting recovery of redox homeostasis. Exogenous NRF2 expression reduced cell death (Fig. 5G) and restored the proliferative ability of TRIB2-depleted HB cells (Fig. 5H, I, Supplementary Fig. 5E, F). Together, these results demonstrate that NRF2 reactivation can effectively compensate for TRIB2 loss by preventing lipid peroxidation and restoring proliferation in HB.

### TCF3 directly binds to TRIB2 regulatory elements and activates its transcription in HB

**Fig. 6:**
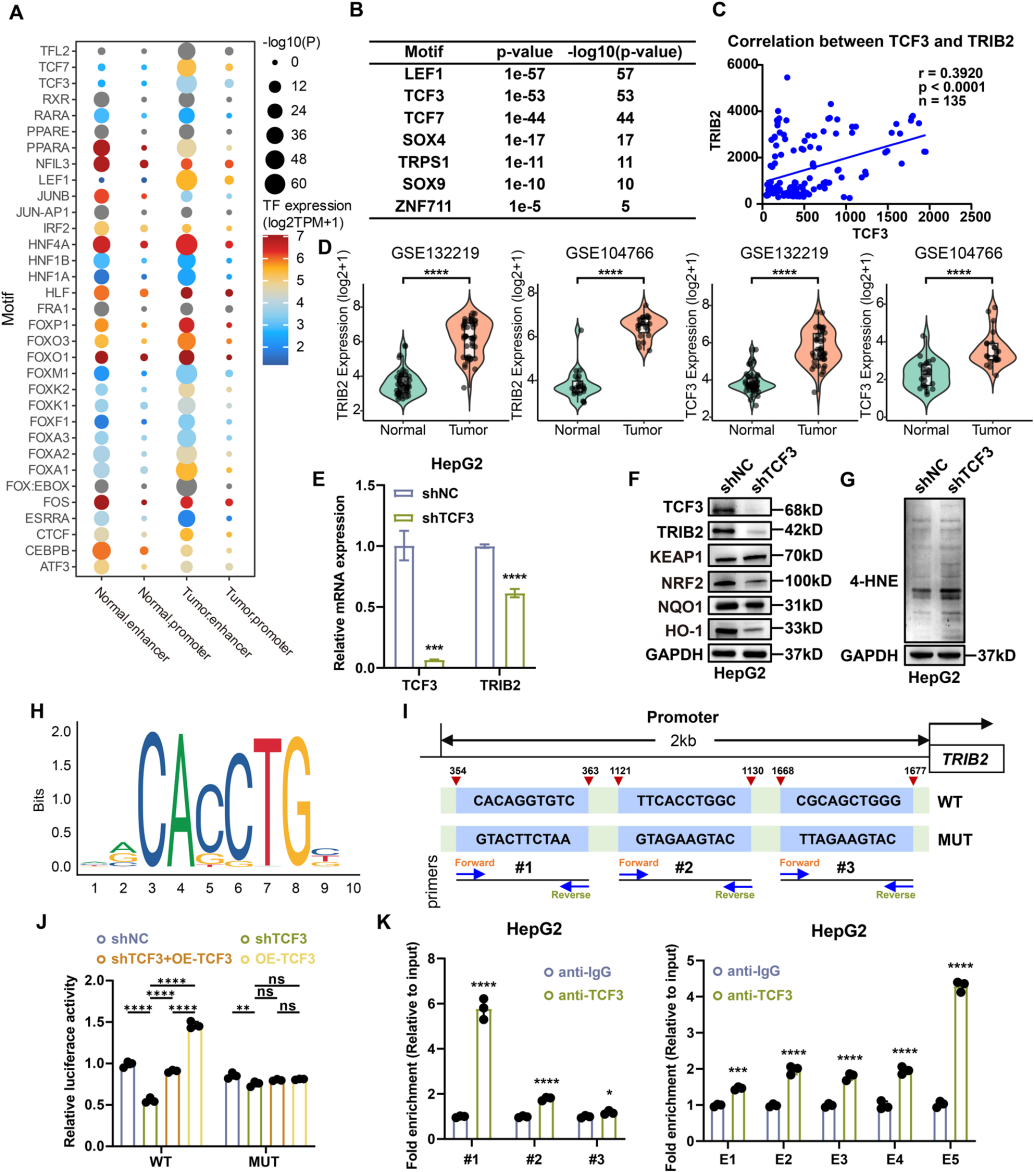
TCF3 binds to TRIB2 regulatory elements and activates transcription. **A** Motif enrichment analysis was performed on tissue-specific promoter and enhancer regions. **B** Intersection of HB-specific promoter motifs and up-regulated genes, showing significant P-values for related TFs. **C** Correlation analysis between TCF3 and TRIB2 expression in HB datasets (GSE132219 and GSE104766). **D** TCF3 and TRIB2 expression in datasets GSE132219 (n = 43 HB and 46 non-tumor samples) and GSE104766 (n = 23 HB and 23 non-tumor samples), respectively. **E** qRT-PCR analysis of TRIB2 in HepG2 cells with or without TCF3 knockdown (n = 3 independent biological replicates). **F**-**G** Western blots of TRIB2, NRF2, HO-1, NQO1 (**F**), and 4-HNE (**G**) in HepG2 cells with or without TCF3 knockdown. **H** Identification of TCF3-binding motif within TRIB2 promoter using JASPAR database. **I** Schematic of the TRIB2 promoter showing TCF3-binding sites and mutation design. **J** Luciferase reporter assays showing TRIB2 promoter activity regulated by TCF3 overexpression, knockdown, or binding site mutation (n = 3 independent biological replicates). **K** ChIP-qPCR assays suggested the occupancy of TCF3 at TRIB2 promoter (left) and enhancer (right) elements (n = 3 independent biological replicates)

TFs are essential regulators of gene expression that function by binding to regulatory elements and controlling E-P interactions [44]. Having demonstrated that HB-specific SEs promote oncogenesis through TRIB2-associated chromatin looping, we sought to identify which TFs contribute to the regulation of TRIB2 E-P activity. Motif enrichment analysis of HB-specific enhancer and promoter regions (Fig. 6A), combined with upregulated transcriptomic profiles, identified LEF1, TCF3, TCF7, SOX4, and SOX9 as top candidate TFs (Fig. 6B, Supplementary Fig. 6A). Among these, TCF3 exhibited notable co-expression correlation with TRIB2 (Fig. 6C, Supplementary Fig. 6B). In addition, both TCF3 and TRIB2 were significantly overexpressed in HB compared with non-tumor tissues across two independent cohorts (GSE132219 and GSE104766; Fig. 6D). Systematic knockdown screening revealed that only TCF3 depletion markedly reduced TRIB2 expression at both mRNA and protein levels (Fig. 6E, F, Supplementary Fig. 6G), whereas silencing of other candidates showed negligible effects (data not shown). Furthermore, TCF3 knockdown significantly suppressed cell proliferation, as shown by CCK-8 and colony formation assays (Supplementary Fig. 6C, D), and enhanced ferroptosis, as indicated by increased lipid ROS levels detected by flow cytometry (Supplementary Fig. 6E), decreased IC50 values for ferroptosis inducers (Supplementary Fig. 6F), elevated 4-HNE protein adducts (Fig. 6G, Supplementary Fig. 6H), and increased cell mortality observed by PI staining (Supplementary Fig. 6I). Motif analysis using the JASPAR database identified three high-confidence TCF3-binding motifs within the TRIB2 promoter region (Fig. 6H). Based on these sites, we constructed wild-type (WT) and mutant (MUT) luciferase reporter plasmids (Fig. 6I). Co-transfection with TCF3 overexpression plasmids significantly enhanced transcriptional activity of the WT promoter, whereas TCF3 knockdown suppressed it, with mutation of three TCF3-binding sites abolishing this induction (Fig. 6J). Among these, the mutation at site 1 (MUT1) exerted the most pronounced effect, suggesting that this site plays a predominant role in mediating the transcriptional response to TCF3 (Supplementary Fig. 6J). ChIP-qPCR confirmed TCF3 occupancy at both the TRIB2 promoter and all enhancer regions, with the strongest enrichment observed at promoter site 1 (Fig. 6K). Collectively, these findings demonstrate that TCF3 directly binds to the TRIB2-SE and promoter, thereby promoting TRIB2 transcriptional activation in HB.

### Clinical correlations and prognostic significance of the TCF3-TRIB2-NRF2 axis in HB

**Fig. 7:**
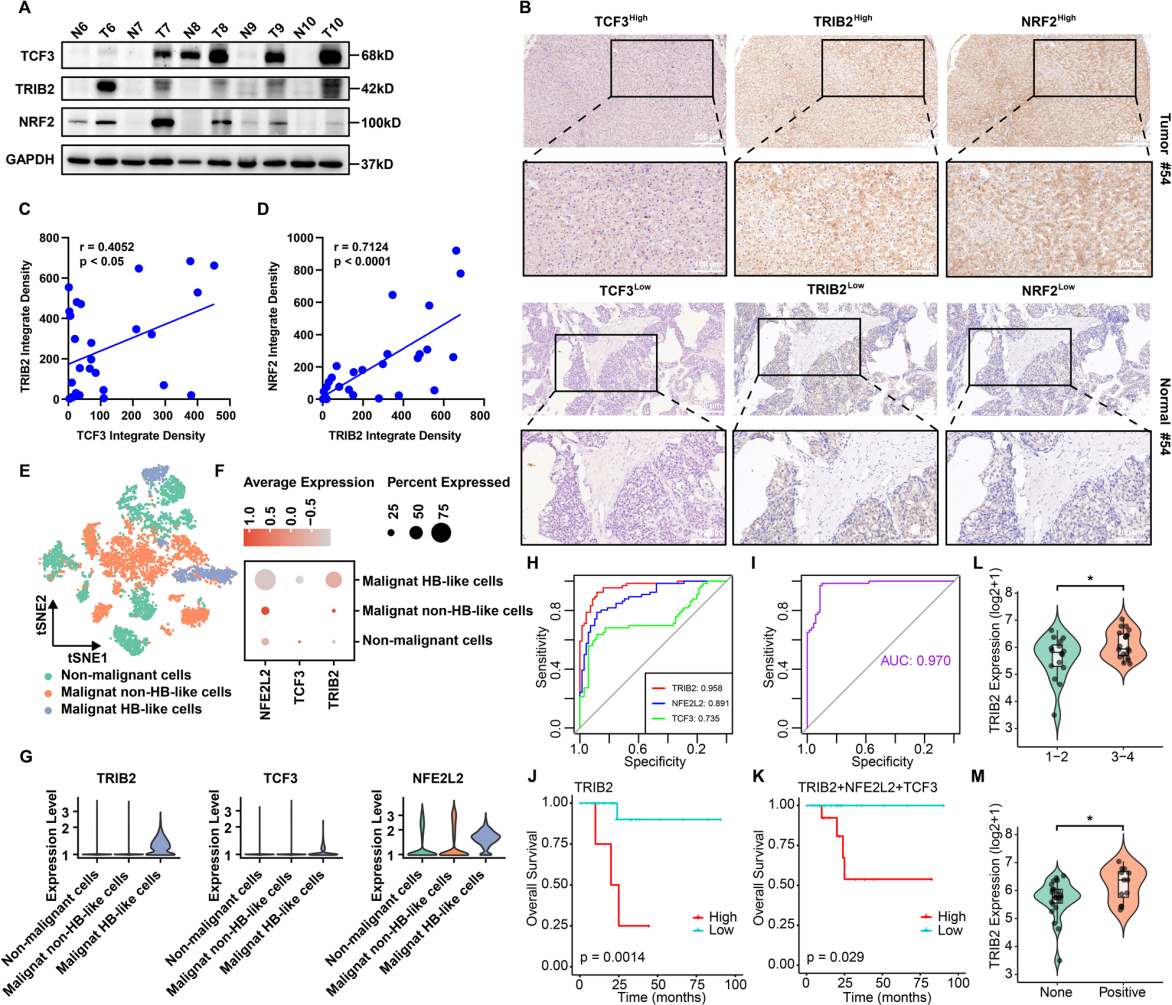
Clinical validation of the TCF3-TRIB2-NRF2 axis in HB. **A** Validation of TRIB2, TCF3, and NRF2 high expression in HB clinical specimens via Western blot (n = 5 paired samples). **B** Representative IHC profiles of TCF3, TRIB2, and NRF2 protein expression in tumor tissue (high; upper panel) and matched adjacent non-tumor tissue (low; lower panel). **C** Correlation analysis between TCF3 and TRIB2 via IHC staining (n = 28 samples). **D** Correlation analysis between TRIB2 and NRF2 via IHC staining (n = 28 samples). **E** t-SNE clustering of hepatocytes from HB (n = 8) and adjacent (n = 6) pediatric liver samples, showing 3 groups in the plot, annotated based on copy number variations and embryonic liver progenitor signatures. **F** Dot plots showing expression levels of TRIB2, TCF3, and NFE2L2 in 3 hepatocyte groups. **G** Violin plots showing expression levels of TRIB2, TCF3, and NFE2L2 in 3 hepatocyte groups. **H**-**I** Diagnostic ROC curves for individual markers (**H**) and TCF3-TRIB2-NFE2L2 combined signature (**I**) in HB datasets (GSE132219 and GSE104766, n = 66 HB and 69 non-tumor samples). **J**-**K** Kaplan-Meier survival curves of overall survival based on TRIB2 expression (**J**) and TCF3-TRIB2-NFE2L2 combined signature (**K**) (n = 35 patients). **L**-**M** TRIB2 expression in HB tissues with patients’ PRETEXT stage (**L**) and metastasis situation (**M**) (n = 35 patients)

To examine the clinical relevance of the TCF3-TRIB2-NRF2 axis, we first confirmed elevated expression of all three proteins in HB tissues compared with non-tumor controls (Fig. 7A). Tissue microarray-based analyses (Fig. 7B) revealed significant co-expression correlations between TCF3 and TRIB2 (Pearson *r* = 0.405, *p* < 0.05; Fig. 7C) and between TRIB2 and NRF2 (Pearson *r* = 0.712, *p* < 0.0001; Fig. 7D). Re-analysis of our previously published HB single-cell RNA sequencing (scRNA-seq) dataset, which classified hepatocytes into three distinct subpopulations [45], showed marked enrichment of TCF3, TRIB2, and NFE2L2 (encoding NRF2 protein) transcripts in the “malignant HB-like cells”. These cells were characterized by high copy number variation burden and elevated stemness gene expression compared with the other two clusters (Fig. 7E-G), suggesting coordinated involvement in HB malignant transformation. For diagnostic evaluation, receiver operating characteristic (ROC) analysis integrating two public HB datasets (GSE132219 and GSE104766) demonstrated strong discriminatory ability in distinguishing HB from non-tumor liver tissues for individual markers (TRIB2 AUC = 0.958; TCF3 AUC = 0.735; NFE2L2 AUC = 0.891; Fig. 7H) and even higher performance for the combined TCF3-TRIB2-NFE2L2 signature (AUC = 0.970; Fig. 7I). Using our unpublished RNA-seq dataset of 35 patients with detailed clinical characteristics (Supplementary Table 5), we confirmed the strong diagnostic performance of both the individual markers (TRIB2 AUC = 0.956; TCF3 AUC = 0.956; NFE2L2 AUC = 0.804; Supplementary Fig. 7A) and the combined three-gene signature (AUC = 0.979; Supplementary Fig. 7B). Regarding prognostic, Kaplan-Meier survival analysis of our cohort revealed significantly reduced overall survival in patients with high TRIB2 expression (Fig. 7J) and in those exhibiting co-elevated expression of TCF3, TRIB2, and NFE2L2 (Fig. 7K). Moreover, TRIB2 overexpression was significantly associated with advanced PRETEXT stages (Fig. 7L) and distant metastasis (Fig. 7M). These results indicate that elevated TCF3-TRIB2-NRF2 expression defines a clinically aggressive HB subtype with strong diagnostic and prognostic significance.

## Discussion

**Fig. 8:**
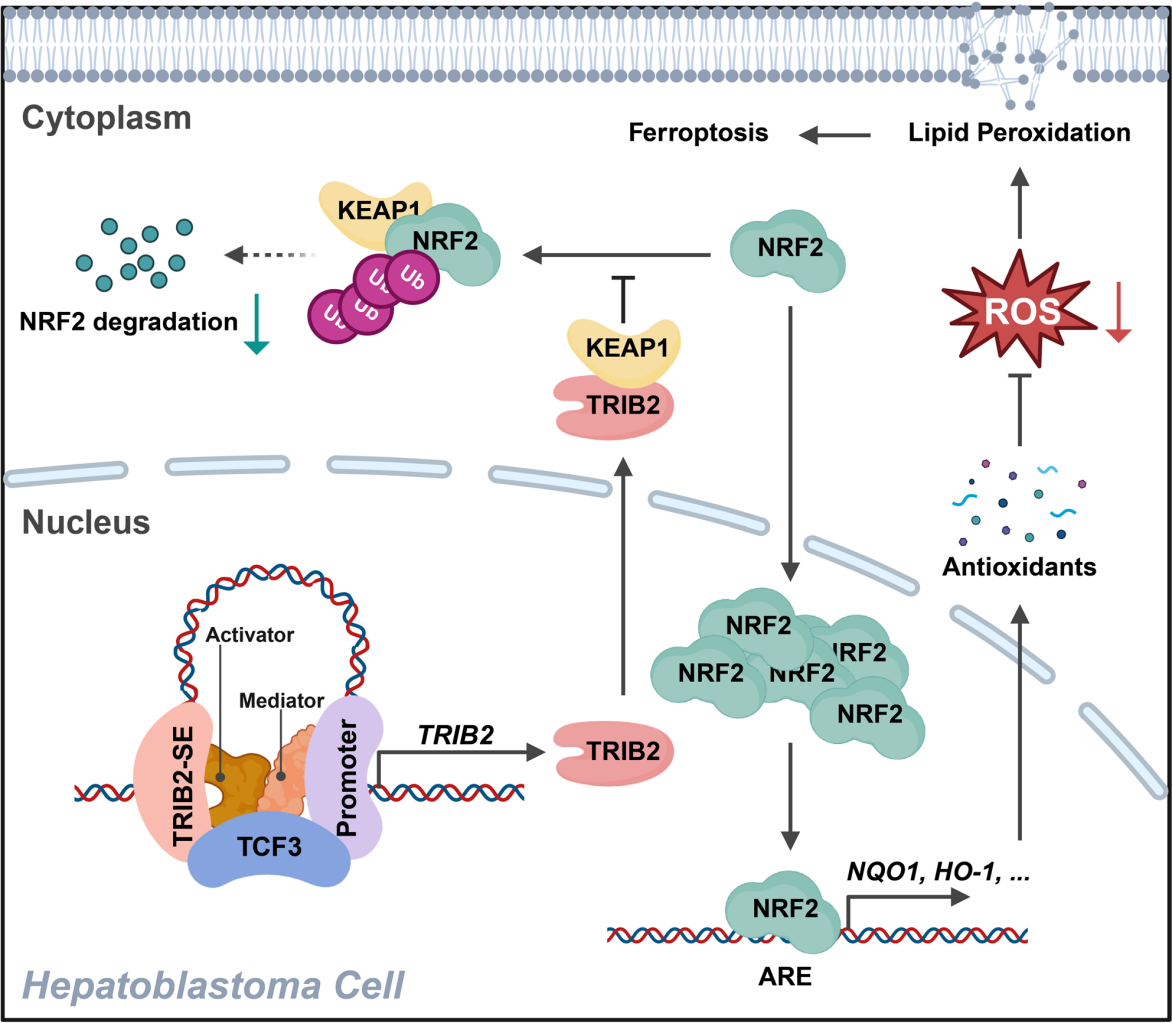
Schematic model of TCF3-promoted TRIB2-SE-driven malignant progression in HB. Created in BioRender.com

Functional E-P interactions, particularly those orchestrated by SEs, are fundamental epigenetic drivers of oncogene activation and tumorigenesis across diverse malignancies [46, 47]​. However, the role of 3D chromatin architecture and SE-mediated transcriptional reprogramming in HB pathogenesis remains largely unexplored. In this study, we present the first comprehensive characterization showing that SE-driven TRIB2 expression is mediated through tumor-specific E-P looping, with TCF3 acting as an activator of this mechanism to drive HB pathogenesis (Fig. 8). Previous studies have reported that key bHLH TFs can promote enhancer-driven transcriptional programs. For example, transcription factor 4 (TCF4) binds to the AJUBA SEs and promoter to promote epithelial-mesenchymal transition (EMT) and metastasis in hepatocellular carcinoma (HCC) [48], while MYCN invades and binds to enhancers to amplify tumor-specific transcriptional outputs in neuroblastoma [49].

Ferroptosis has emerged as a pivotal therapeutic vulnerability in cancers, characterized by iron-dependent lipid peroxidation that disrupts redox homeostasis. This process is counteracted by antioxidant defense systems, most notably the KEAP1-NRF2 pathway, which transcriptionally activates cytoprotective genes such as NQO1 and HO-1 to detoxify lipid peroxides and suppress cytotoxic ROS accumulation [50]. Within this context, TRIB2 functions as a critical regulator of ferroptotic sensitivity. While a previous study by Guo et al. showed that TRIB2 reduces ferroptosis in HCC by promoting βTrCP-mediated ubiquitination and degradation of TFRC, thereby limiting the LIP required for lipid peroxidation [51], our findings reveal a distinct HB-specific mechanism. TRIB2 competitively binds to KEAP1, disrupting the KEAP1-NRF2 interaction and stabilizing NRF2, which in turn amplifies antioxidant responses (Fig. 8). This mechanistic divergence underscores two tumor-type-specific functions of TRIB2: regulation of iron metabolism through TFRC degradation in HCC, and control of redox homeostasis via the KEAP1-NRF2 pathway in HB. Functionally, although TRIB2 canonically coordinates ubiquitin-proteasome activity as an E3 adaptor ​ [22], its non-canonical, E3-independent binding to KEAP1 directly connects 3D epigenomic remodeling with ferroptosis resistance in HB.

Clinically, the TCF3-TRIB2-NRF2 axis exhibits strong prognostic and therapeutic relevance in HB. Its components display distinct co-expression in HB tissues and are selectively enriched in malignant HB-like hepatocytes at the single-cell level, indicating their oncogenic significance. Importantly, high expression of TRIB2 or the overall TCF3-TRIB2-NRF2 axis predicts poor overall survival and demonstrates exceptional diagnostic accuracy, highlighting this axis as a potential biomarker for prognostic risk stratification and differential diagnosis. From a therapeutic perspective, our results suggest promising strategies for future research. Prior studies have shown that combined treatment with BET inhibitors and ferroptosis inducers can overcome apoptosis resistance in B-cell lymphoma [52]. This finding supports the potential of exploring a similar synergistic therapeutic approach in HB.

Our multi-omics integration based on clinically annotated HB tissue samples, including Hi-C, H3K27ac CUT&Tag, and ATAC-seq, provides direct in vivo evidence of 3D chromatin reprogramming, representing a major strength over studies limited to cell-line models. Although each assay was performed using two technical replicates, the high reproducibility of the data and the strong consistency across epigenetic layers provide robust validation of our conclusions. The main limitation lies in the single-patient origin of the tissue sample, which necessitates cautious interpretation regarding intertumoral heterogeneity. Future investigations should expand multi-omics profiling to larger clinical cohorts of HB to confirm the generality of the TCF3-TRIB2-NRF2 axis and to identify potential molecular subtypes.

​In conclusion, we delineate a coherent oncogenic framework in which SE-driven TRIB2 expression is activated by TCF3, leading to NRF2 stabilization that suppresses ferroptosis and promotes tumor proliferation in HB. This work establishes how 3D epigenomic reprogramming cooperates with redox adaptation to drive HB pathogenesis, positioning the TCF3-TRIB2-NRF2 axis as a promising biomarker and therapeutic target in HB.

## Supplementary Information

Supplementary Material 1.

Supplementary Material 2.

Supplementary Material 3.

## Data Availability

The raw sequence data reported in this paper have been deposited in the Genome Sequence Archive (Genomics, Proteomics & Bioinformatics 2021) in the National Genomics Data Centre (Nucleic Acids Res 2022), China National Centre for Bioinformation/Beijing Institute of Genomics, Chinese Academy of Sciences (GSA: HRA012154).

## Abbreviations

HB: Hepatoblastoma
SE: Super-enhancer
TF: Transcription factor
ARE: Antioxidant response element
H3K27ac: Histone 3 lysine 27 acetylation
H3K4me1: Histone 3 lysine 4 monomethylation
HNSCC: Head and neck squamous cell carcinoma
SCC: Squamous cell carcinoma
TCF3: Transcription factor 3
bHLH: Basic helix-loop-helix
TRIB2: Tribbles homolog 2
TPM: Transcripts Per Million
DAR: Differentially accessible region
PC1: Principal component 1
TAD: Topologically associating domain
IS: Insulation Score
E-P: Enhancer-Promoter
ABC: Activity-by-Contact
FBS: Fetal bovine serum
CHX: Cycloheximide
siRNA: Small interfering RNA
PBS: Phosphate-buffered saline
qRT-PCR: Real-time quantitative polymerase chain reaction
rRNA: Ribosomal RNA
IP: Immunoprecipitation
BSA: Bovine serum albumin
4-HNE: 4-Hydroxynonenal
IHC: Immunohistochemistry
CRISPRi: CRISPR interference
sgRNA: Short guide RNA​​
ChIP: Chromatin immunoprecipitation
WT: Wild-type
MUT: Mutant
ROS: Reactive oxygen species
PI: Propidium iodide
MDA: Malondialdehyde
GSH: Glutathione
SD: Standard deviation
CUT&Tag: Cleavage Under Targets and Tagmentation
ATAC-seq: Assay for Transposase-Accessible Chromatin using sequencing
DEG: Differentially expressed gene
KEGG: Kyoto Encyclopedia of Genes and Genomes
PRC2: Polycomb repressive complex 2
ECM: Extracellular matrix
RNA Pol II: RNA polymerase II
LIP: Labile iron pool
scRNA-seq: Single-cell RNA-seq
ROC: Receiver operating characteristic
TCF4: Transcription factor 4
EMT: Epithelial-mesenchymal transition
HCC: Hepatocellular carcinoma
