# Supplementary material for: TCF3 activates super-enhancer-driven TRIB2 overexpression to suppress ferroptosis and promote hepatoblastoma proliferation
Source: J Exp Clin Cancer Res. 2025 Dec 29;44:329. doi: 10.1186/s13046-025-03587-1 (PMC12750601; doi:10.1186/s13046-025-03587-1)

Figure 2D

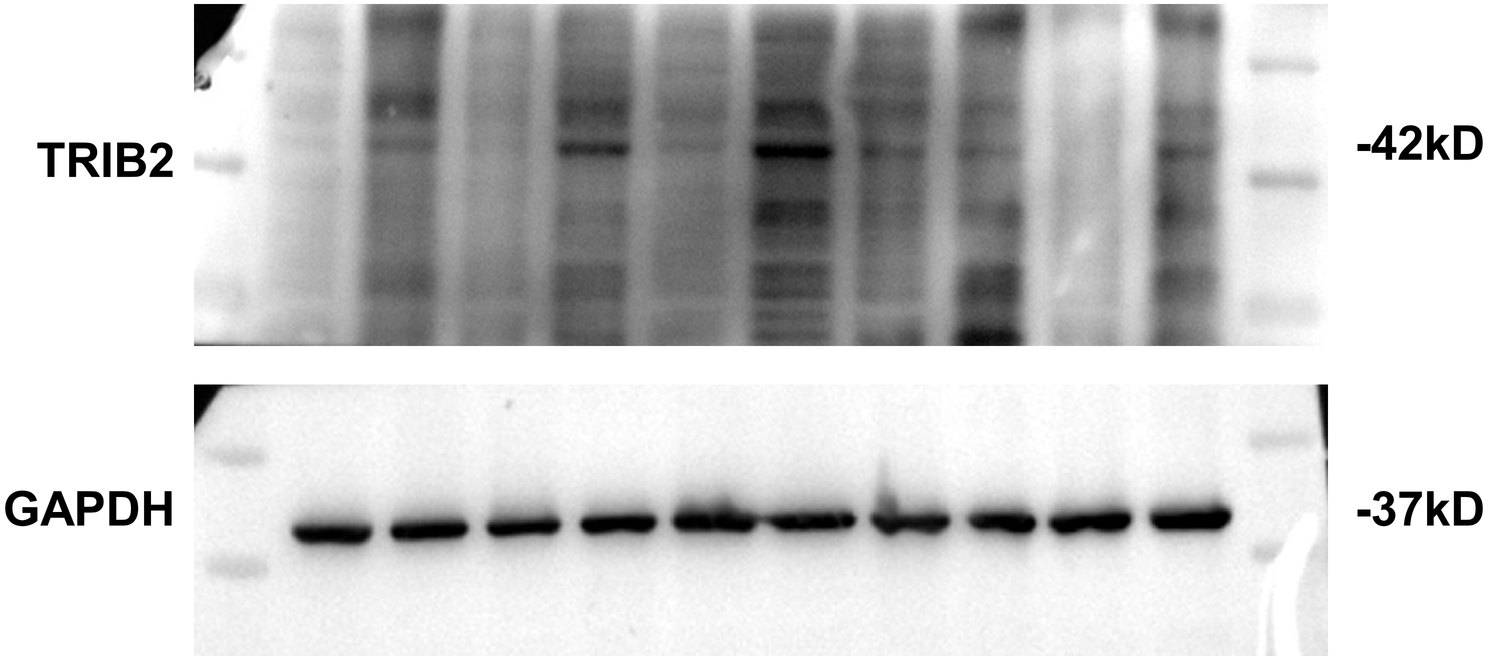

Figure 3G

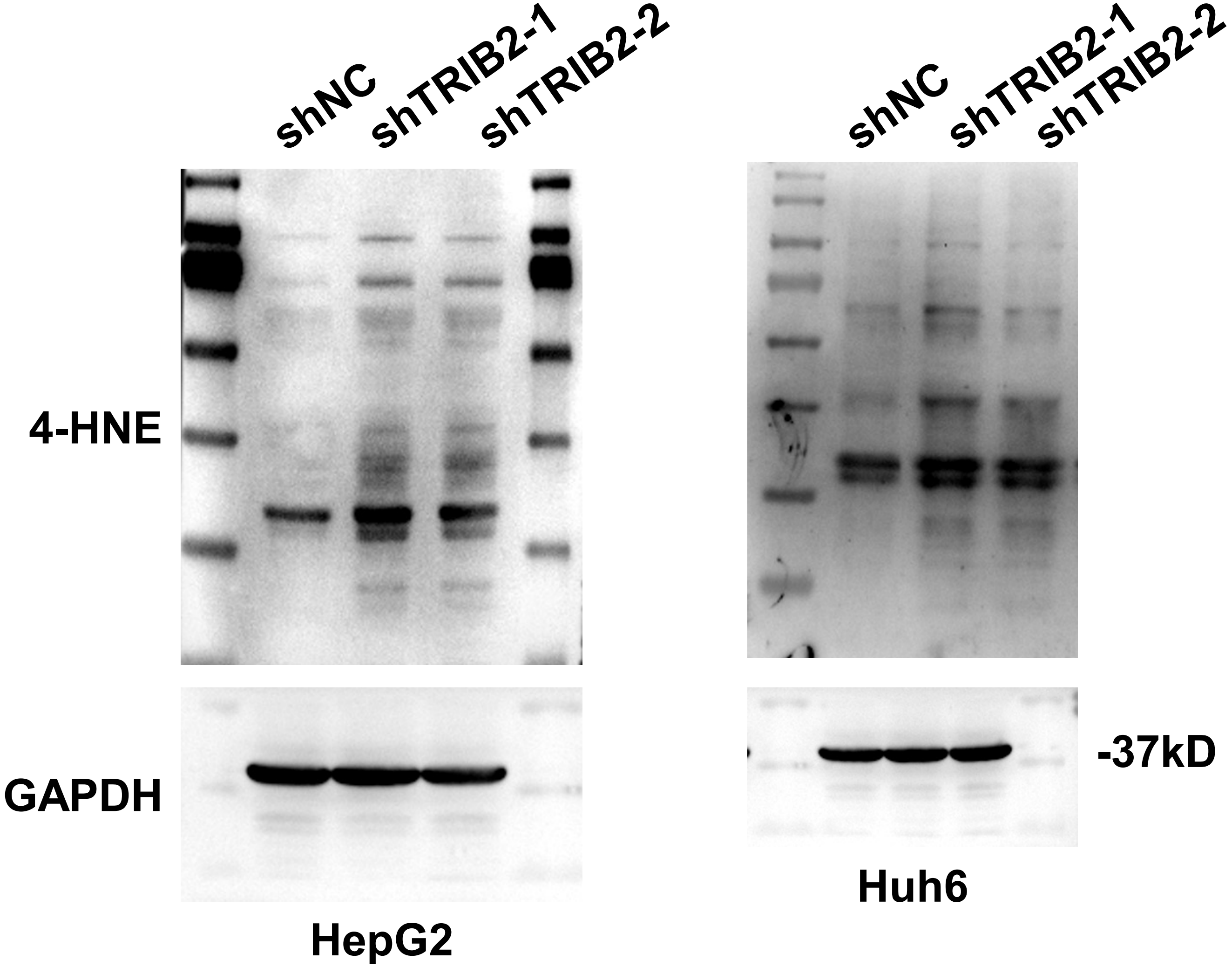
shNC
shTRIB2-1
shTRIB2-2
shNC
shTRIB2-1
shTRIB2-2
4-HNE
GAPDH
-37kD
HepG2
Huh6

Figure 4A

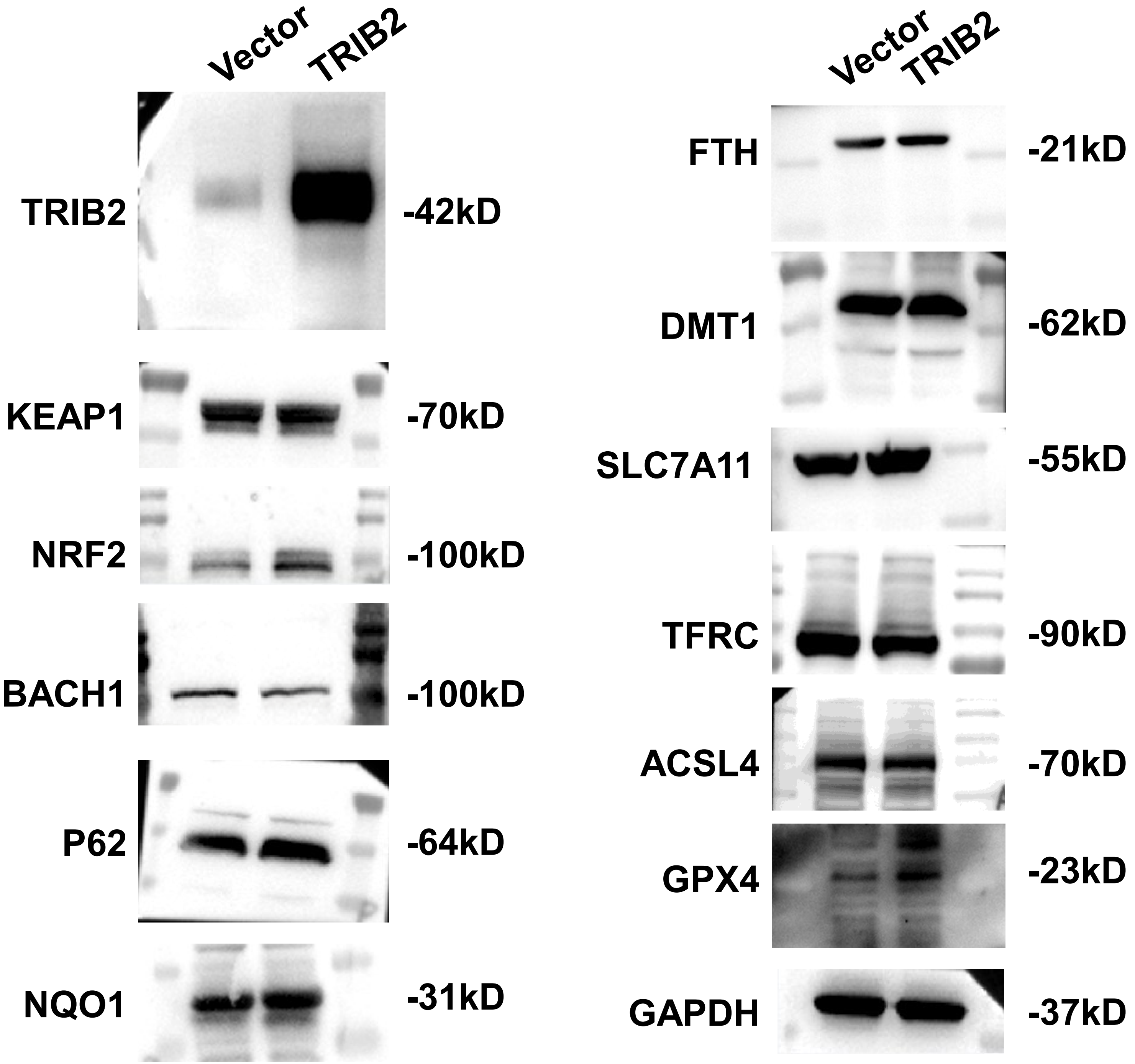
Vector
TRIB2
TRIB2
-42kD
KEAP1
-70kD
NRF2
-100kD
BACH1
-100kD
P62
-64kD
NQO1
-31kD
Vector
TRIB2
FTH
-21kD
DMT1
-62kD
SLC7A11
-55kD
TFRC
-90kD
ACSL4
-70kD
GPX4
-23kD
GAPDH
-37kD

Figure 4D

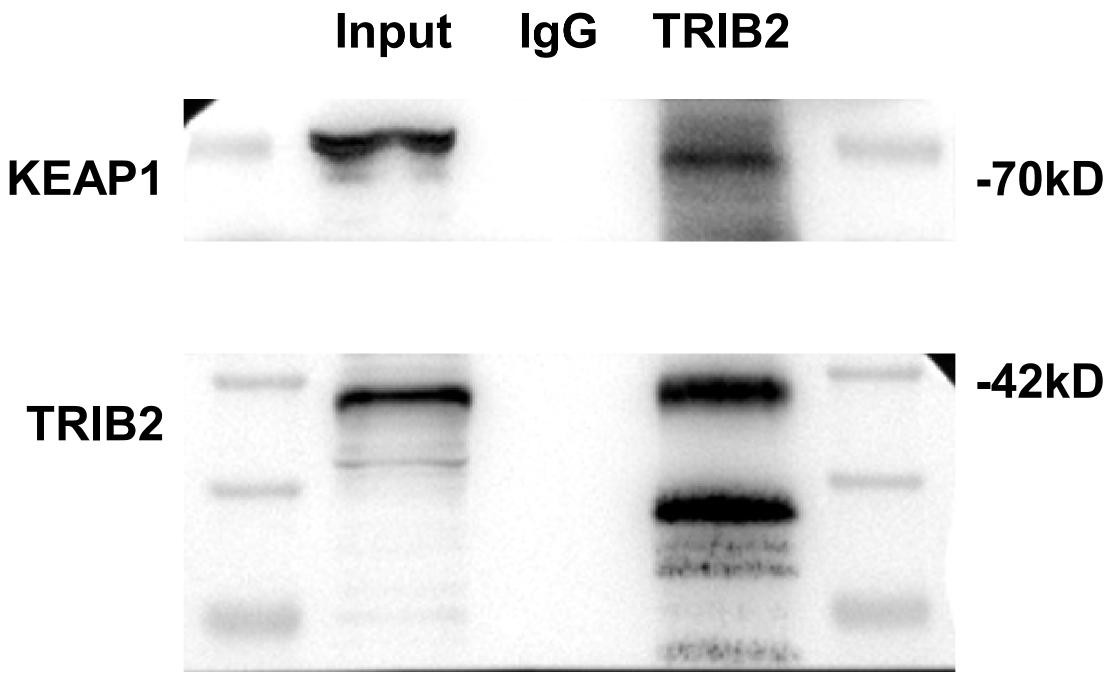

Figure 4E

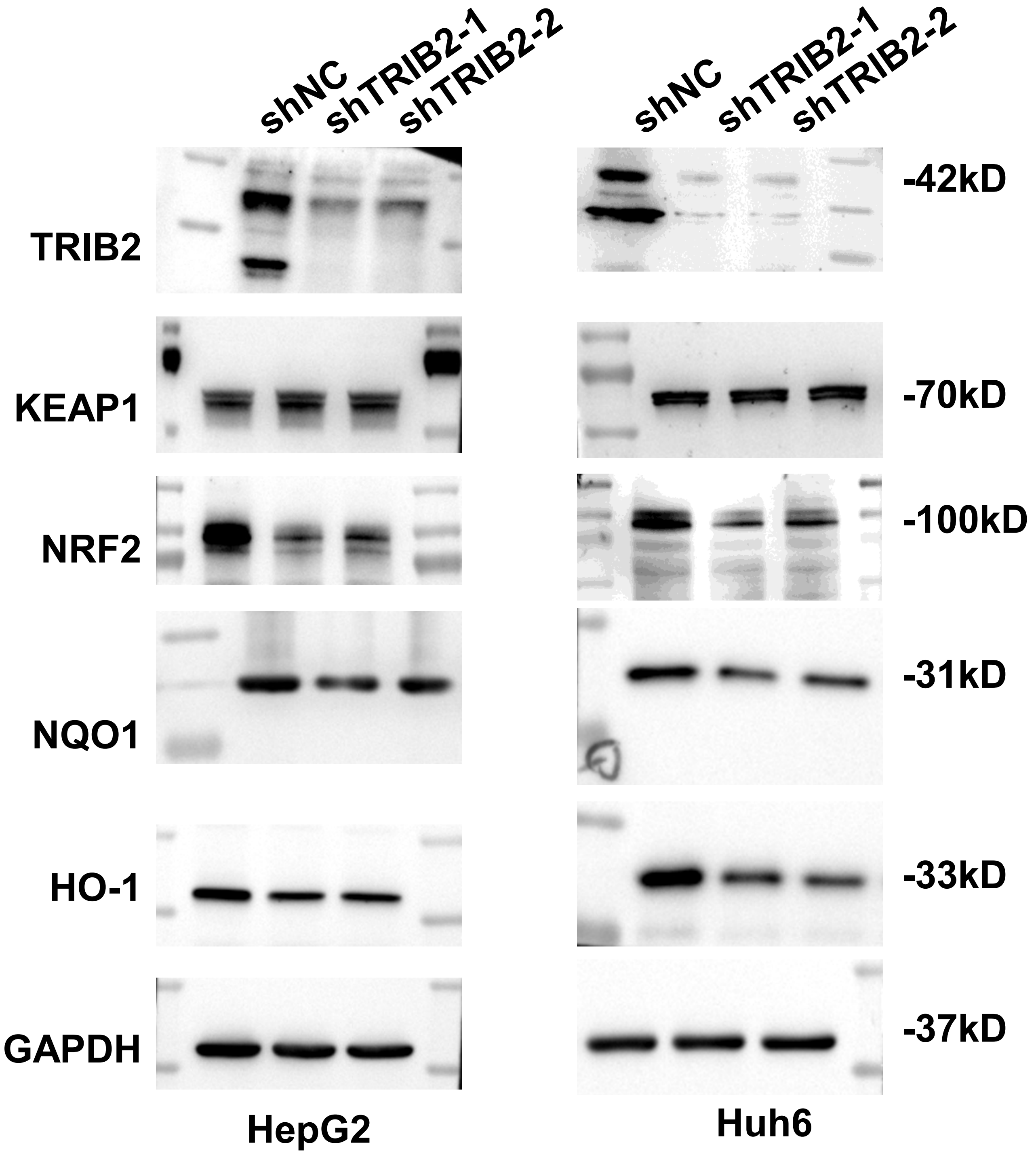
shNC
shTRIB2-1
shTRIB2-2
shNC
shTRIB2-1
shTRIB2-2
TRIB2
KEAP1
NRF2
NQO1
HO-1
GAPDH
-42kD
-70kD
-100kD
-31kD
-33kD
-37kD
HepG2
Huh6

Figure 4H

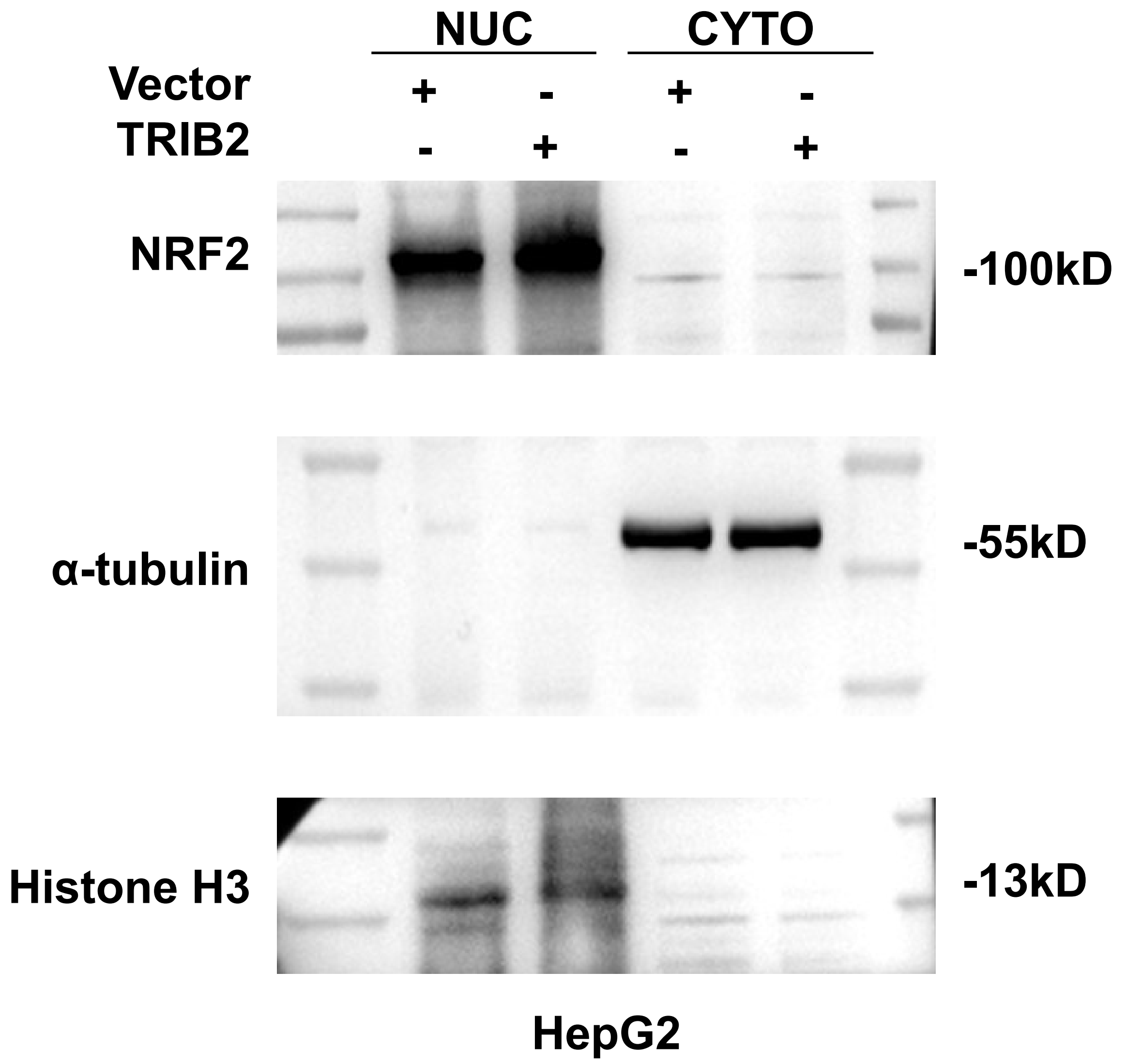
NUC
CYTO
Vector
+
-
+
-
TRIB2
-
+
-
+
NRF2
-100kD
α-tubulin
-55kD
Histone H3
-13kD
HepG2

Figure 4I

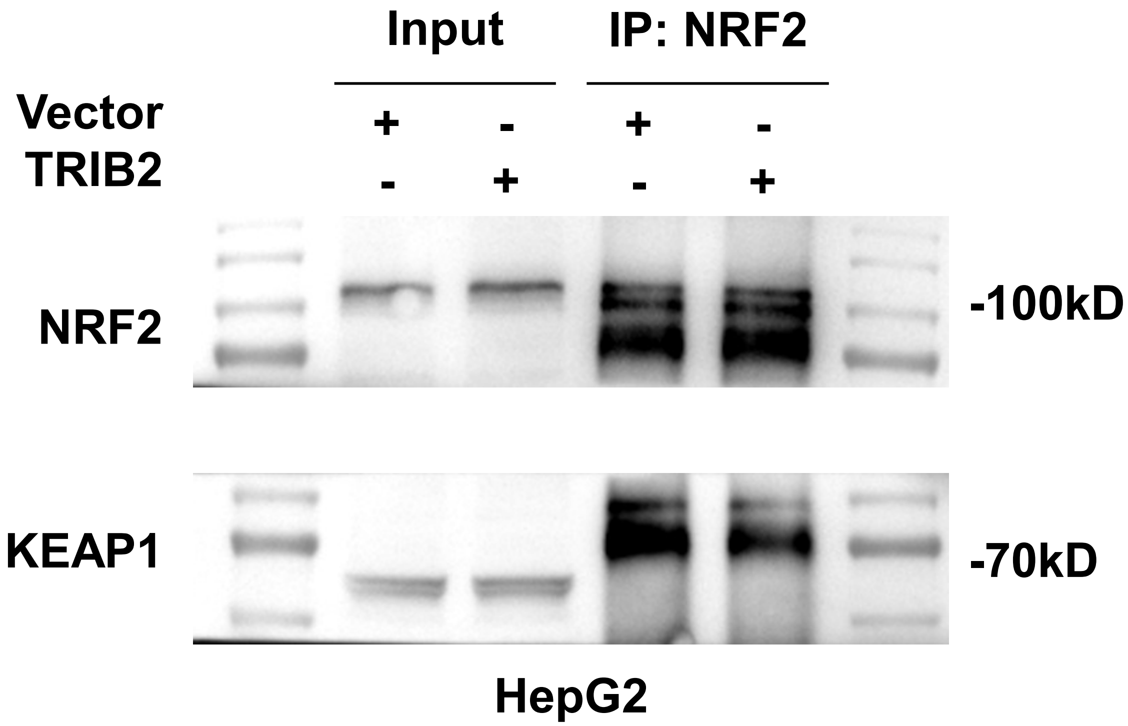

Figure 4J

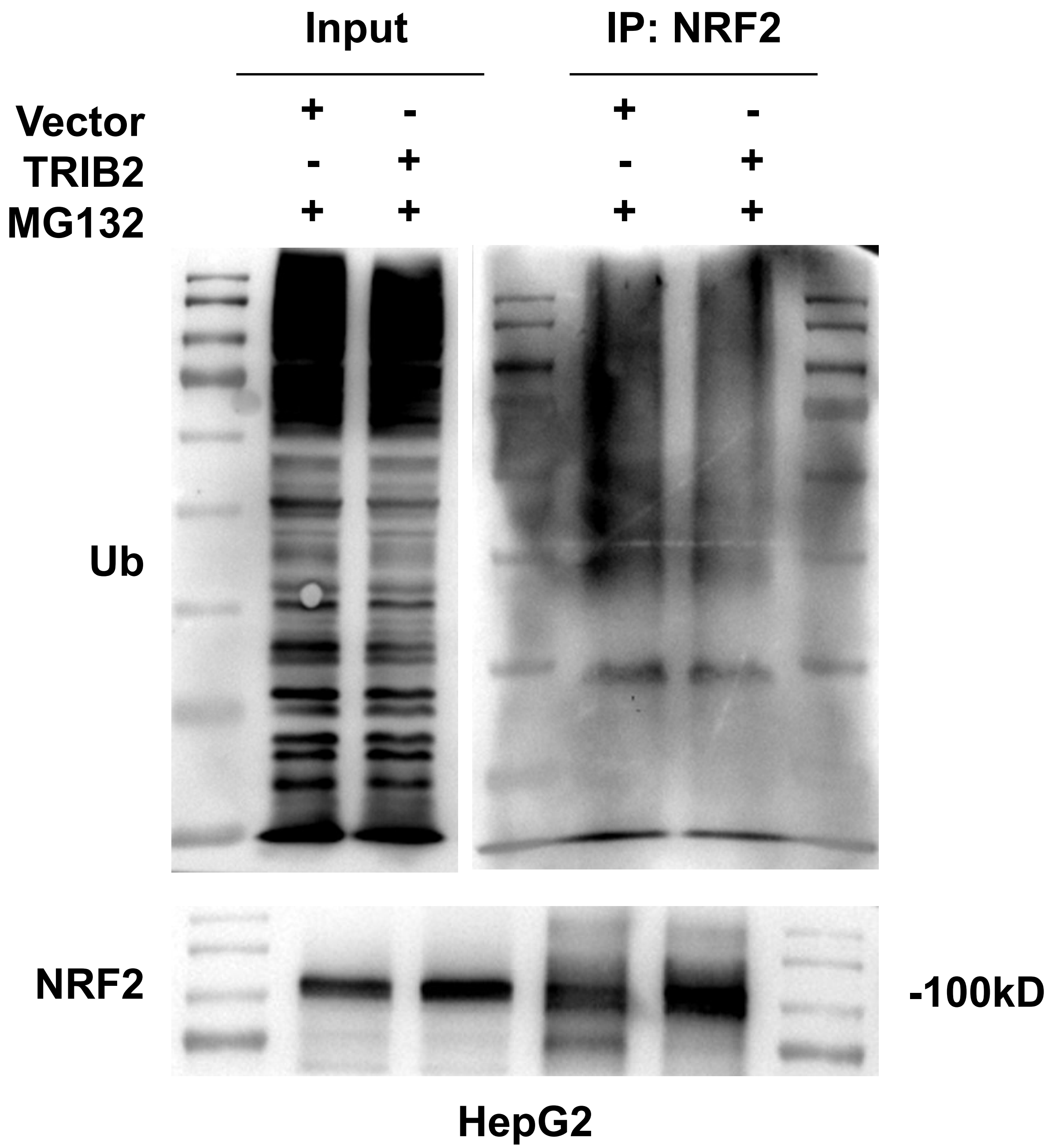

Figure 4K

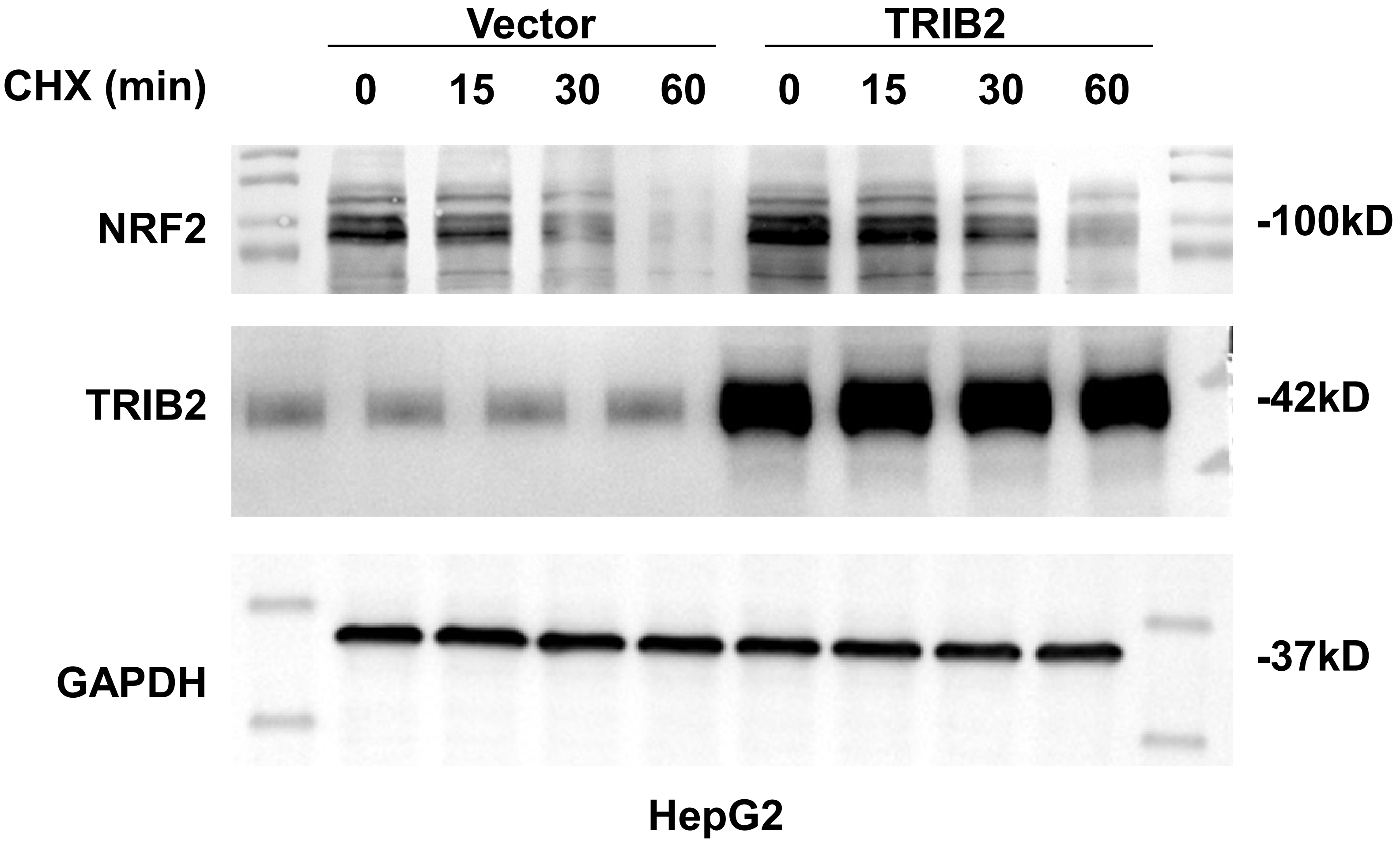
Vector
TRIB2
CHX (min)
0
15
30
60
0
15
30
60
NRF2
-100kD
TRIB2
-42kD
GAPDH
-37kD
HepG2

Figure 5A

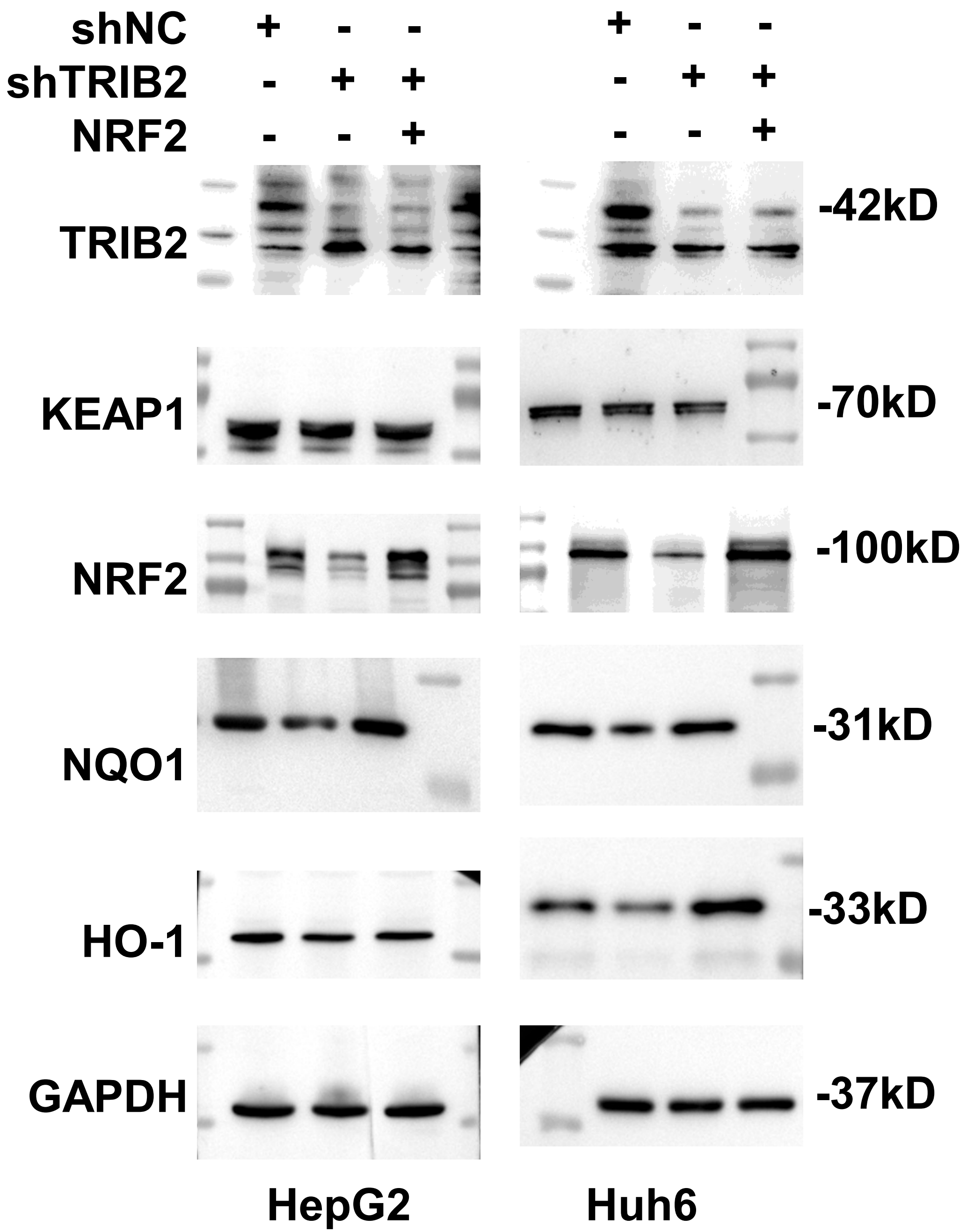

shNC + - - + - -
shTRIB2 - + + - + +
NRF2 - - + - - +
TRIB2
KEAP1
NRF2
NQO1
HO-1
GAPDH
-42kD
-70kD
-100kD
-31kD
-33kD
-37kD
HepG2
Huh6

Figure 5C

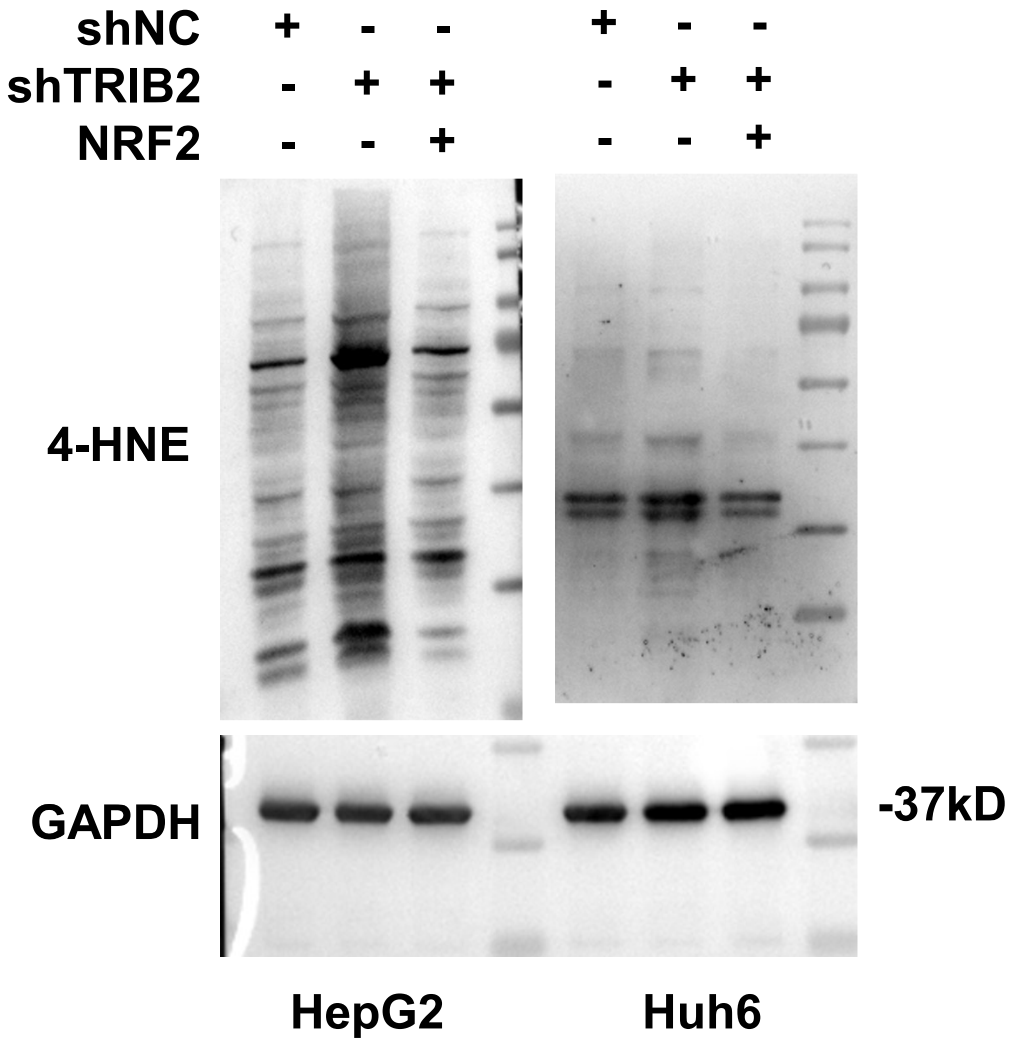

Figure 6F

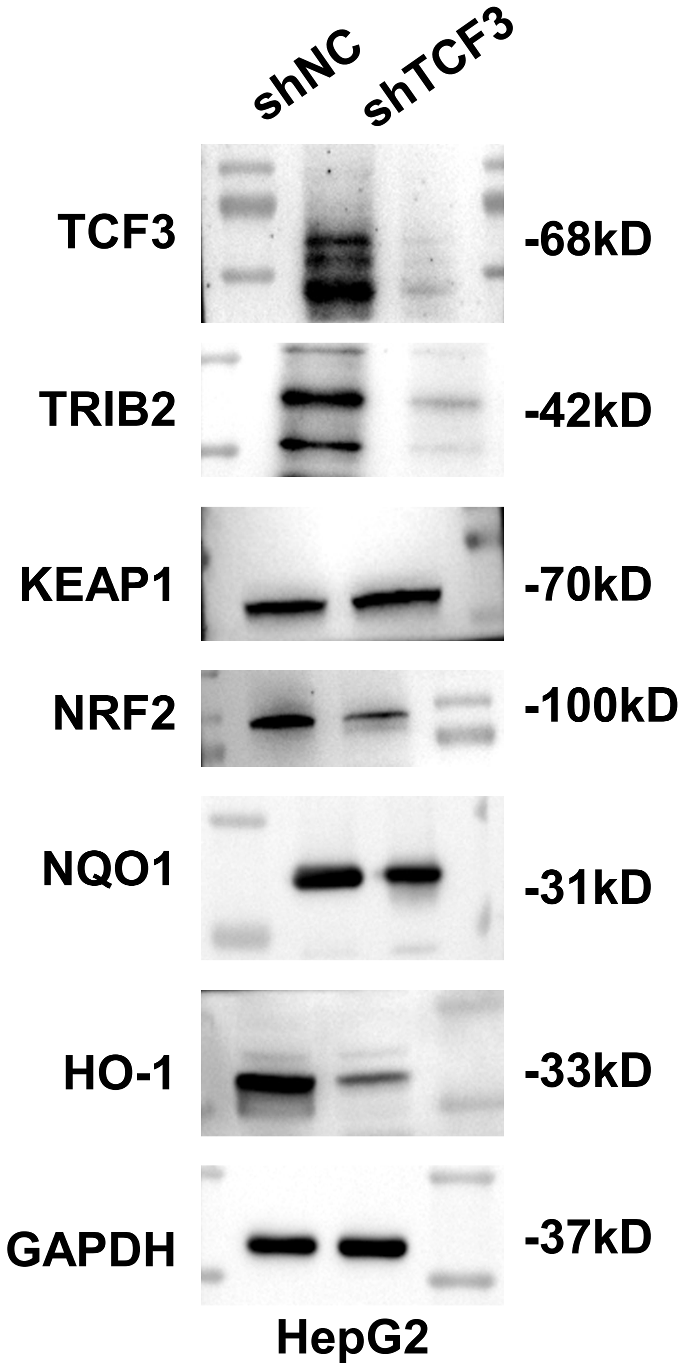
shNC
shTCF3
TCF3
-68kD
TRIB2
-42kD
KEAP1
-70kD
NRF2
-100kD
NQO1
-31kD
HO-1
-33kD
GAPDH
-37kD
HepG2

Figure 6G

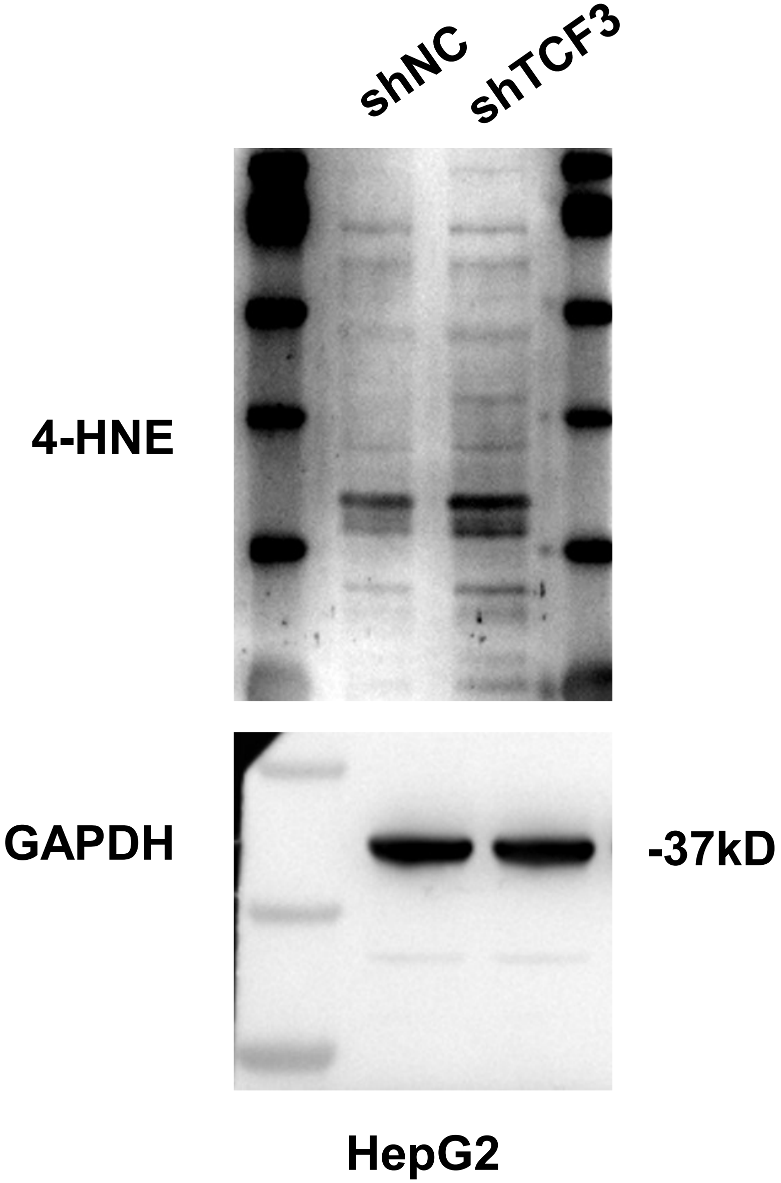

Figure 7A

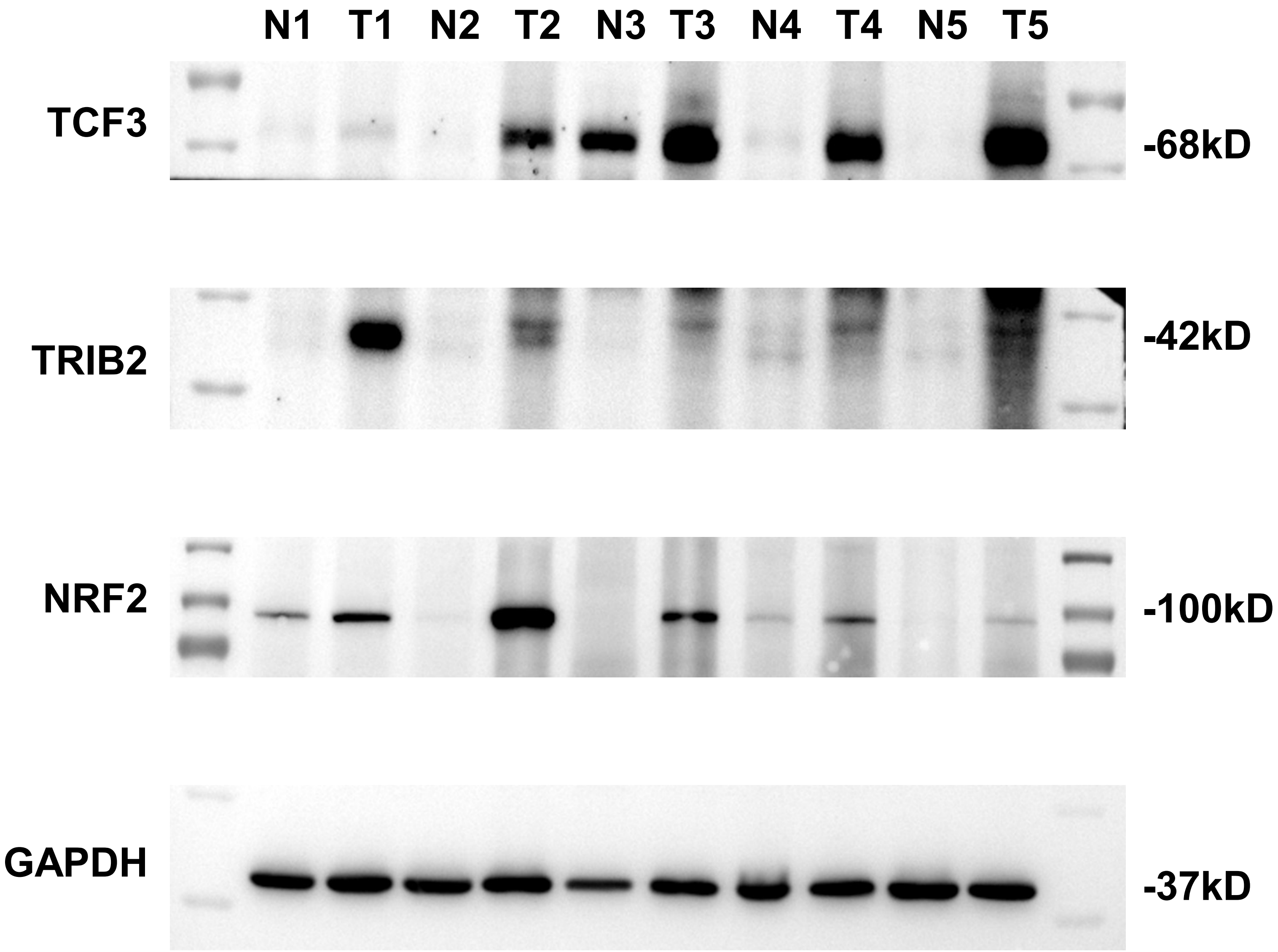
N1 T1 N2 T2 N3 T3 N4 T4 N5 T5
TCF3
-68kD
TRIB2
-42kD
NRF2
-100kD
GAPDH
-37kD

Supplementary Figure 4A

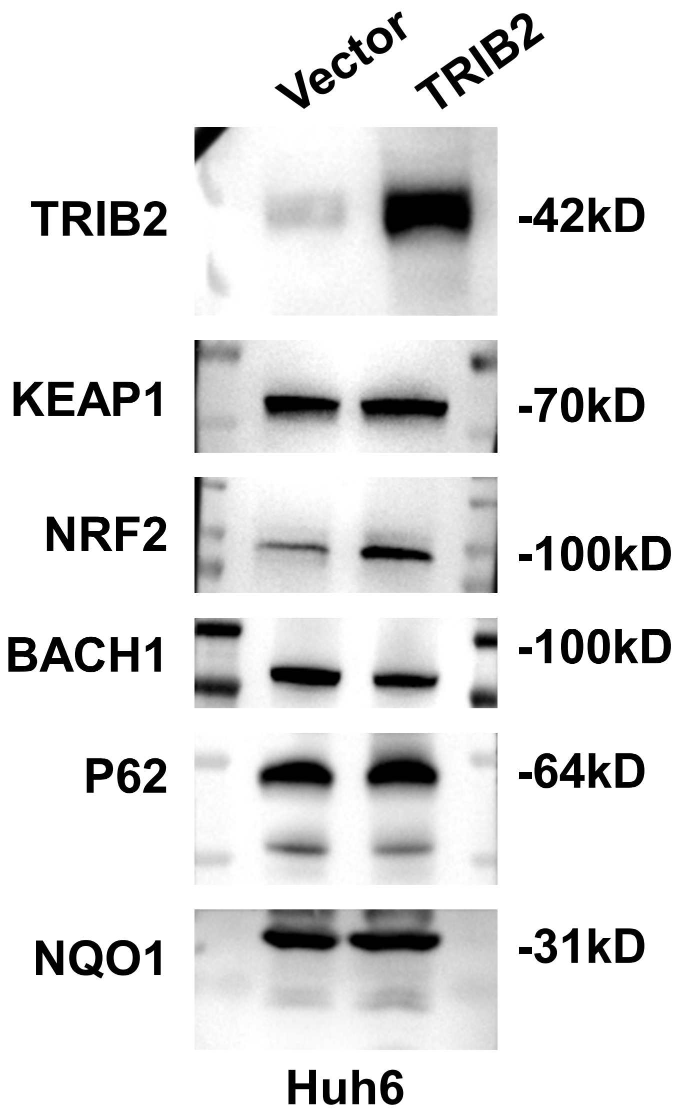

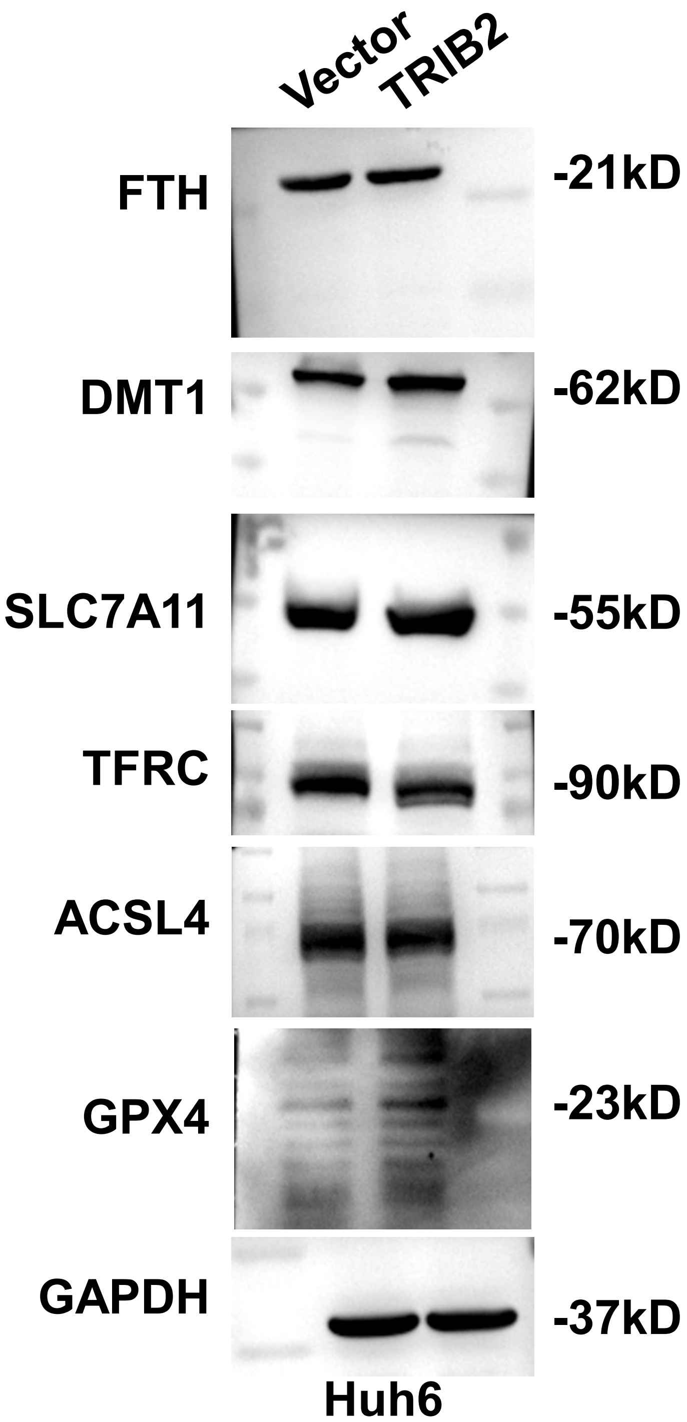

Supplementary Figure 4C

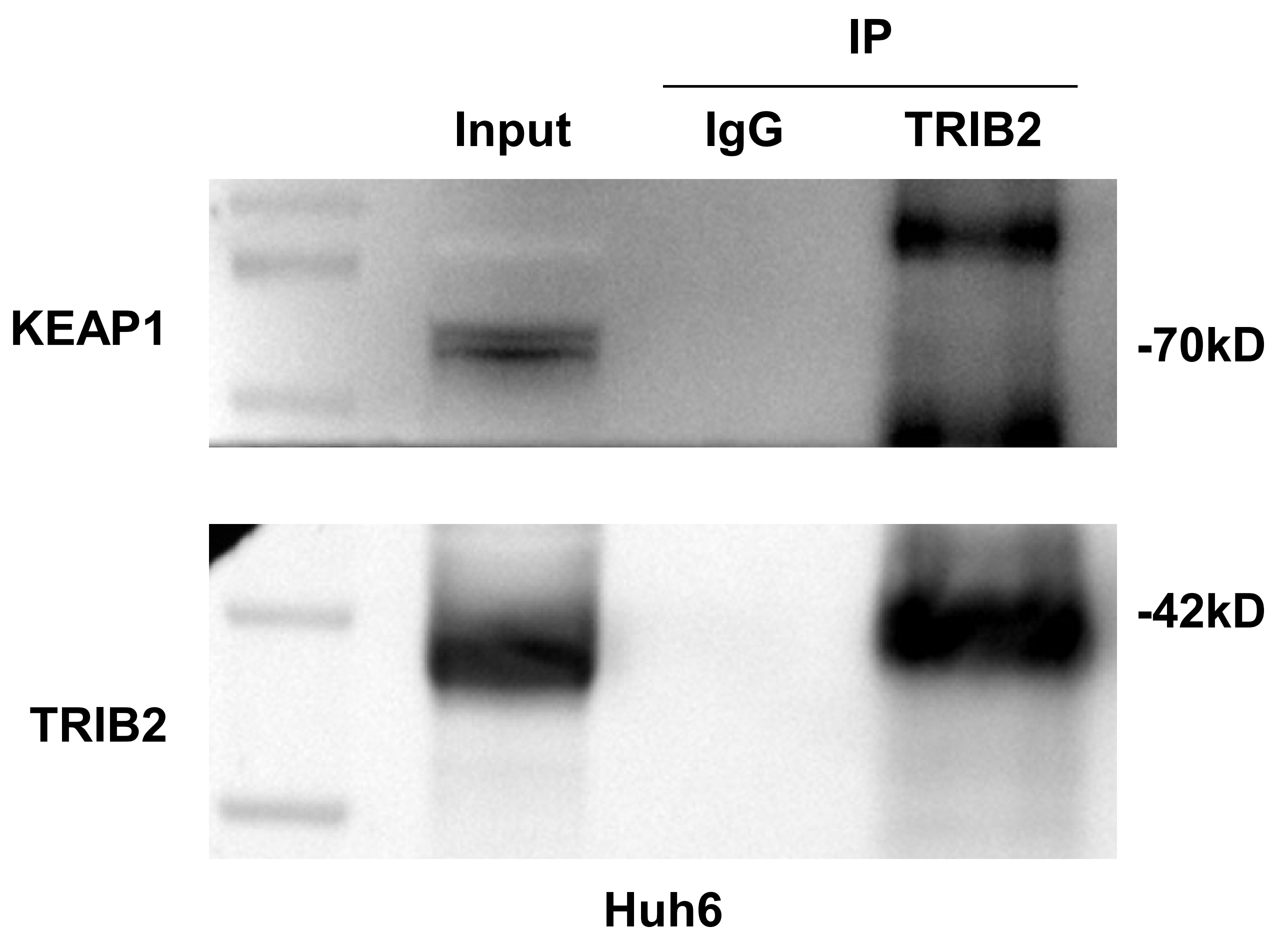

Supplementary Figure 4E

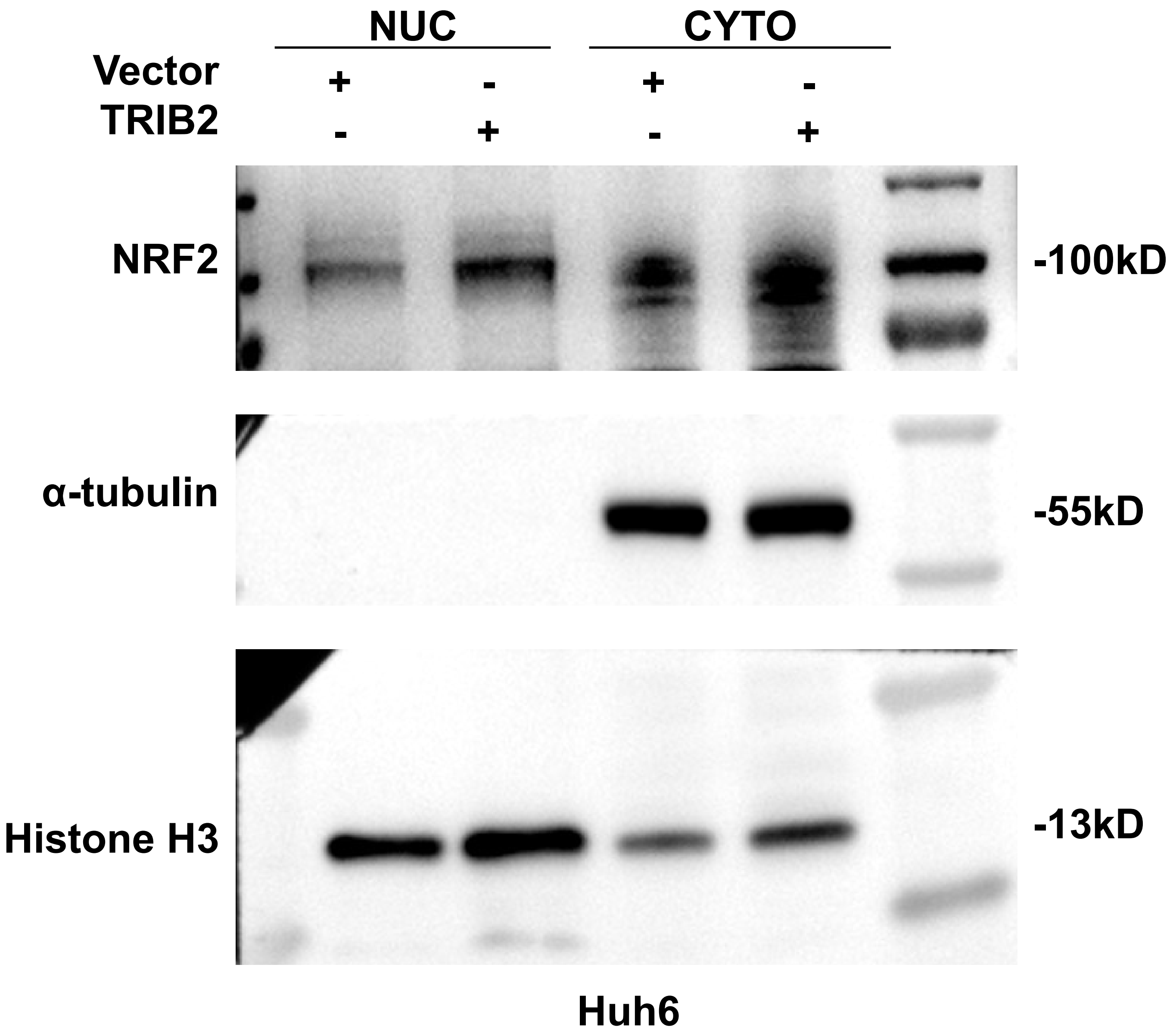

Supplementary Figure 4F

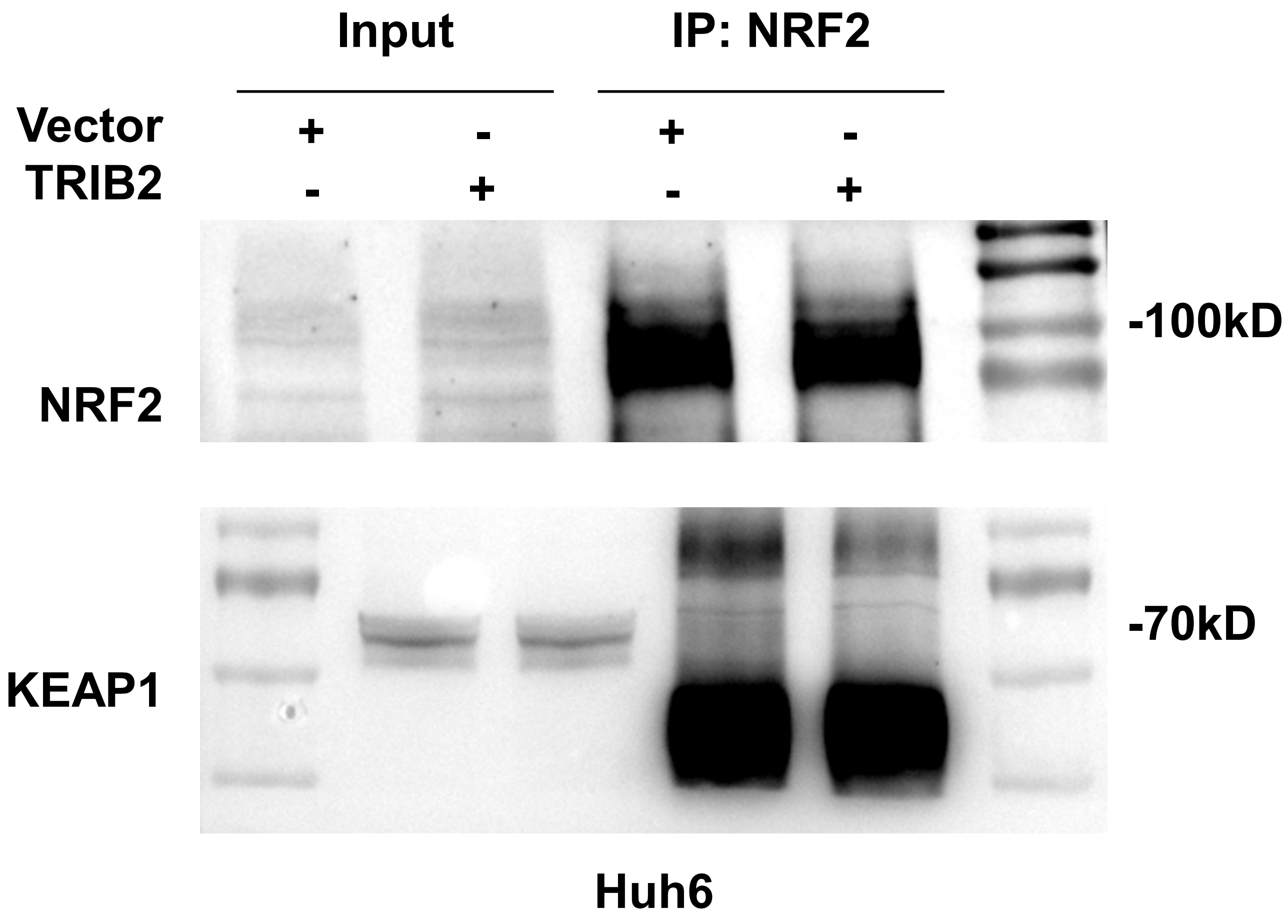

Supplementary Figure 4G

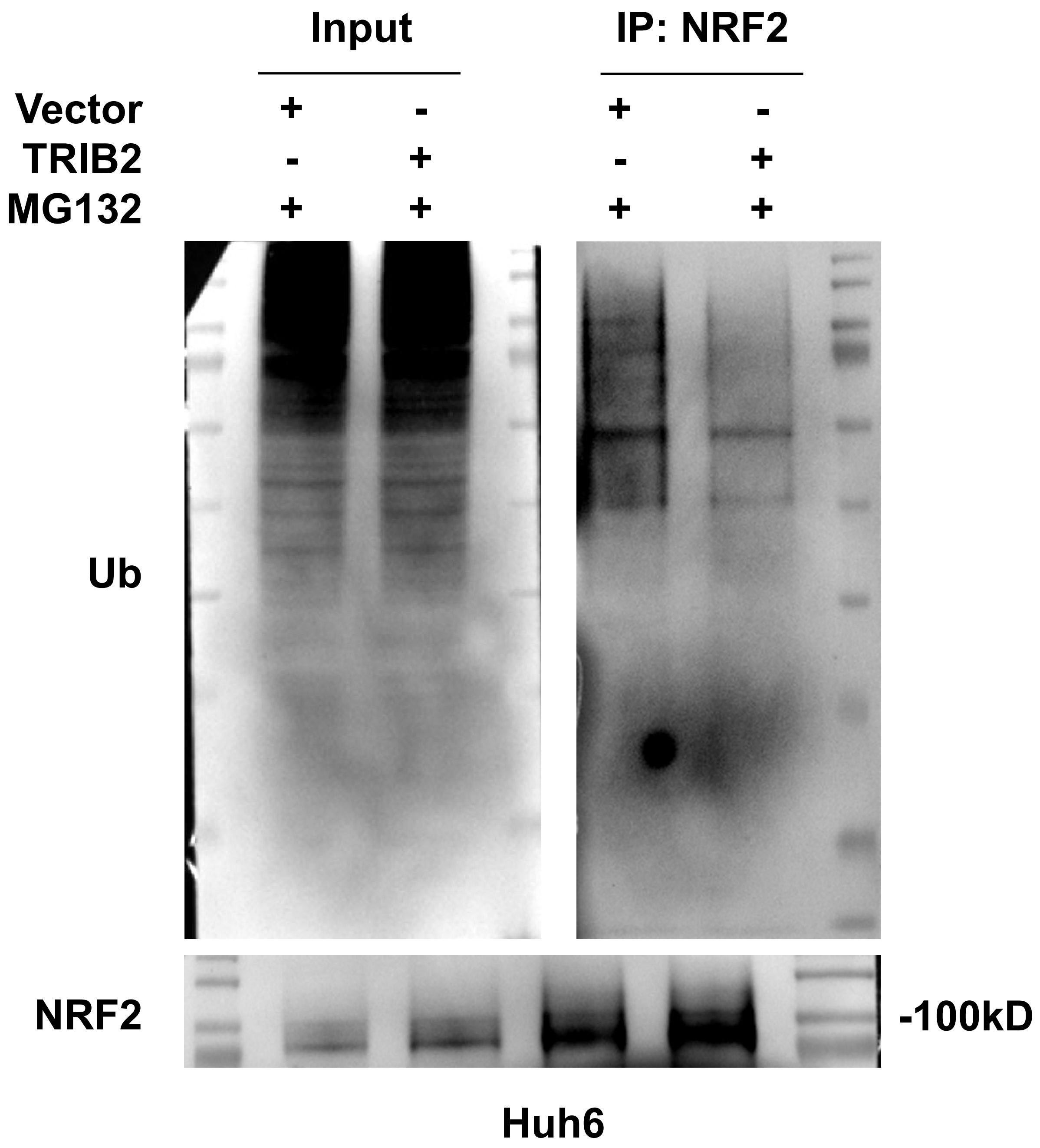

Supplementary Figure 4H

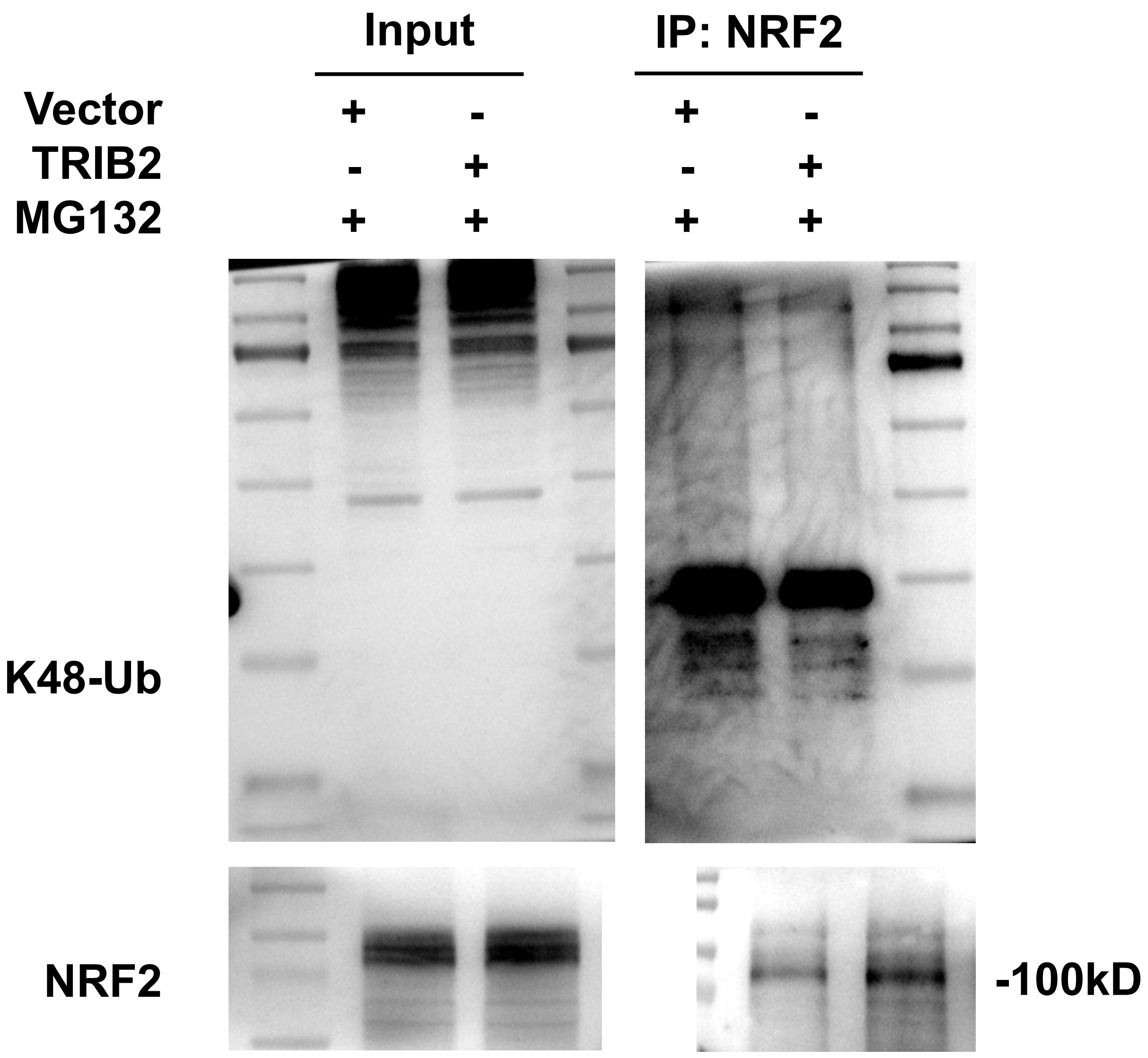

Supplementary Figure 4I

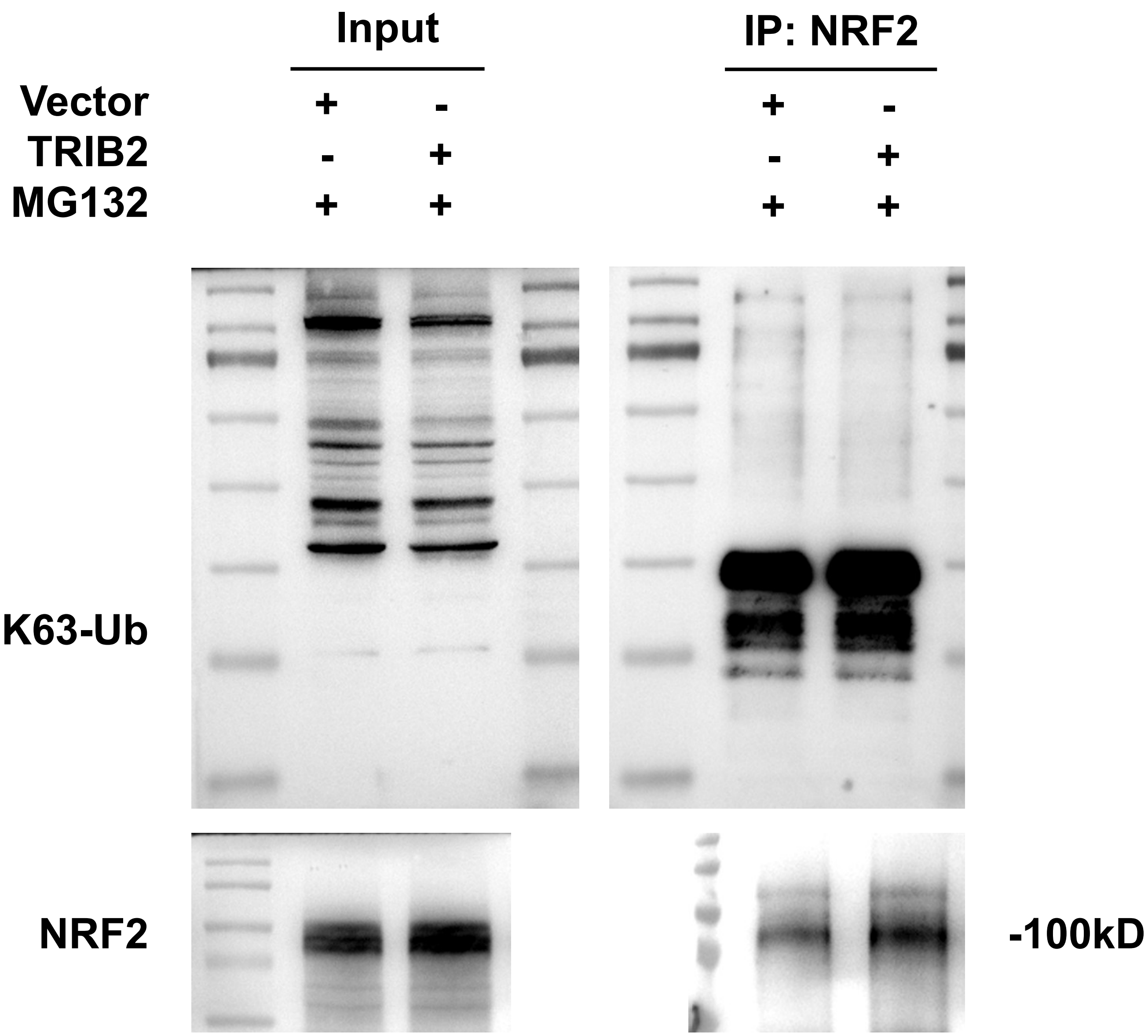

Supplementary Figure 4J

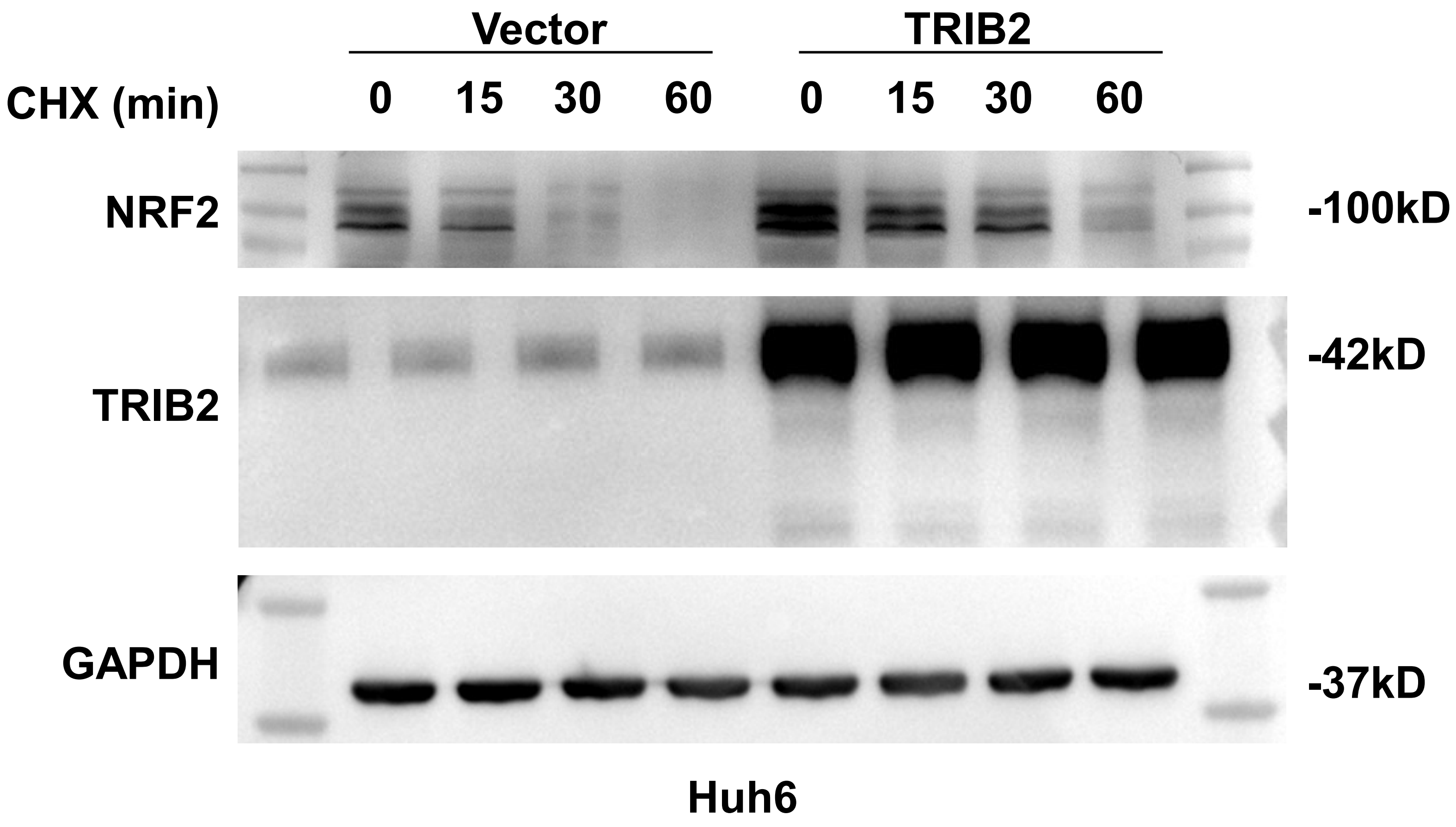

Supplementary Figure 5A

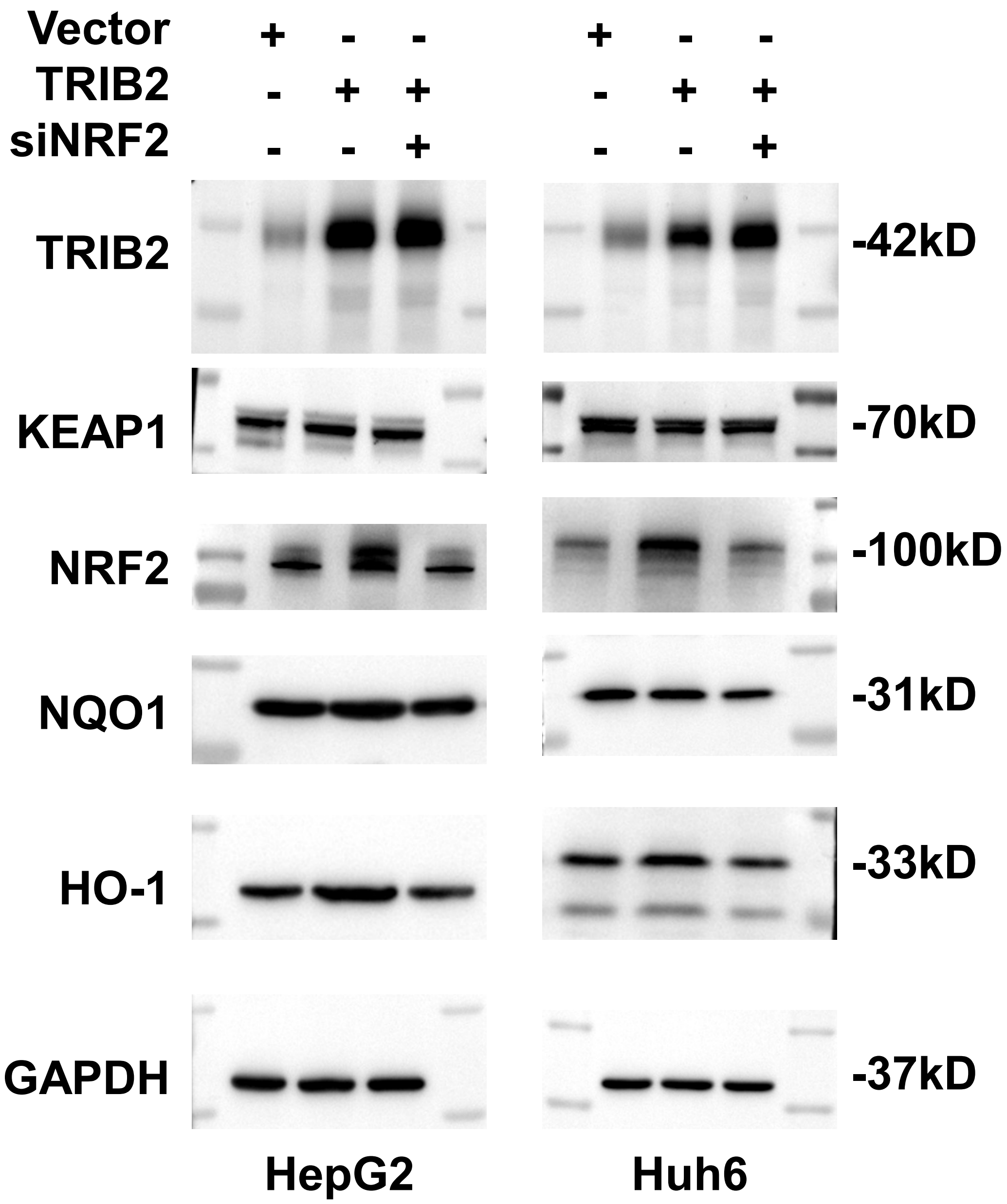

Supplementary Figure 5B

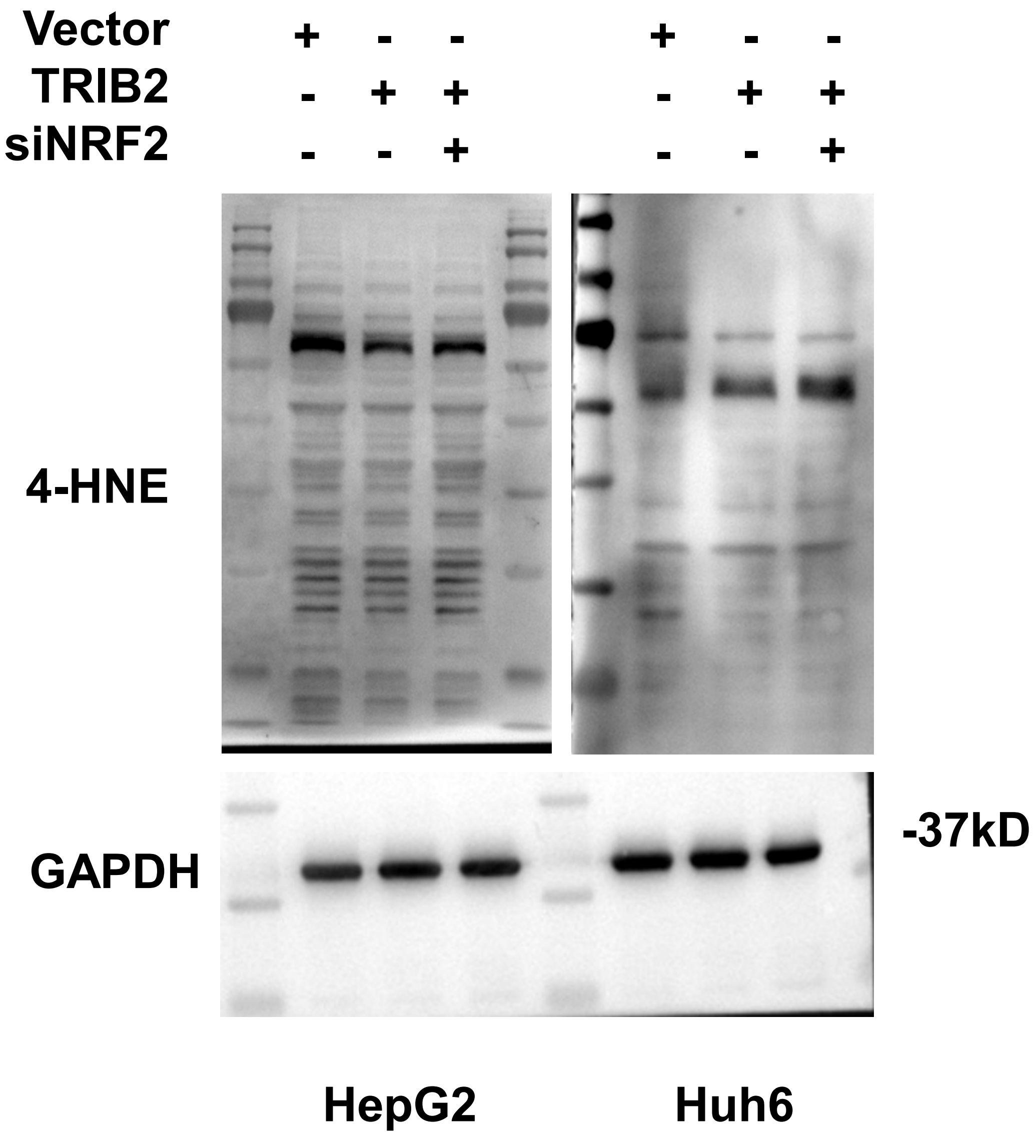

Supplementary Figure 6G

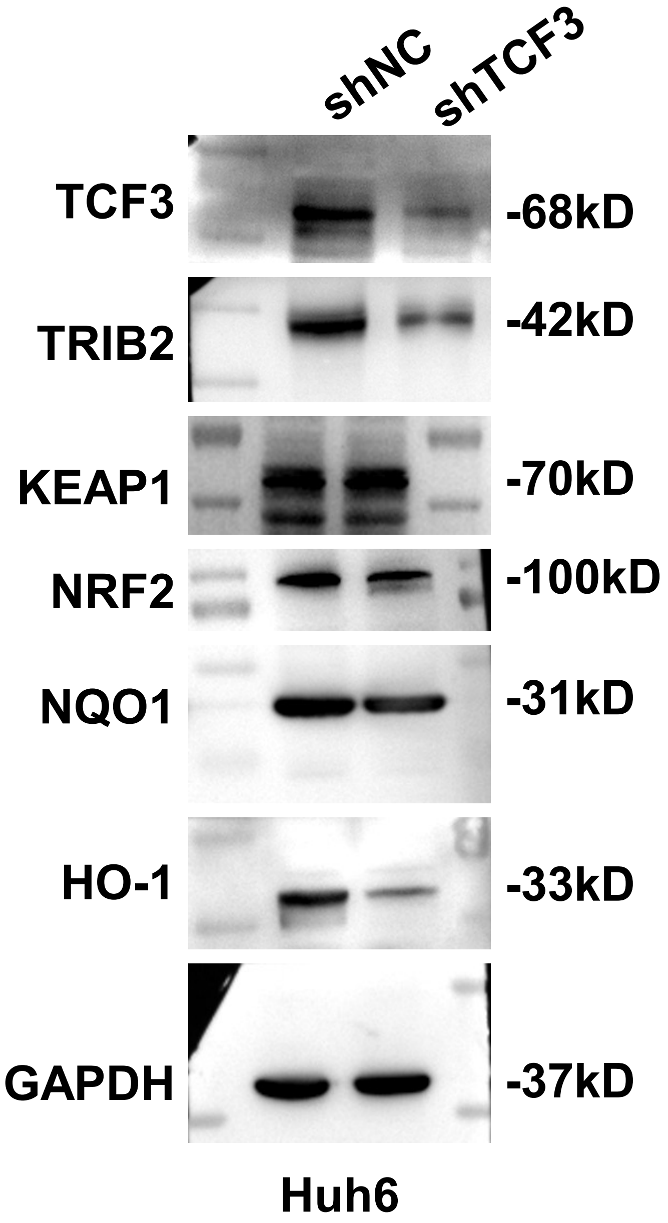

Supplementary Figure 6H

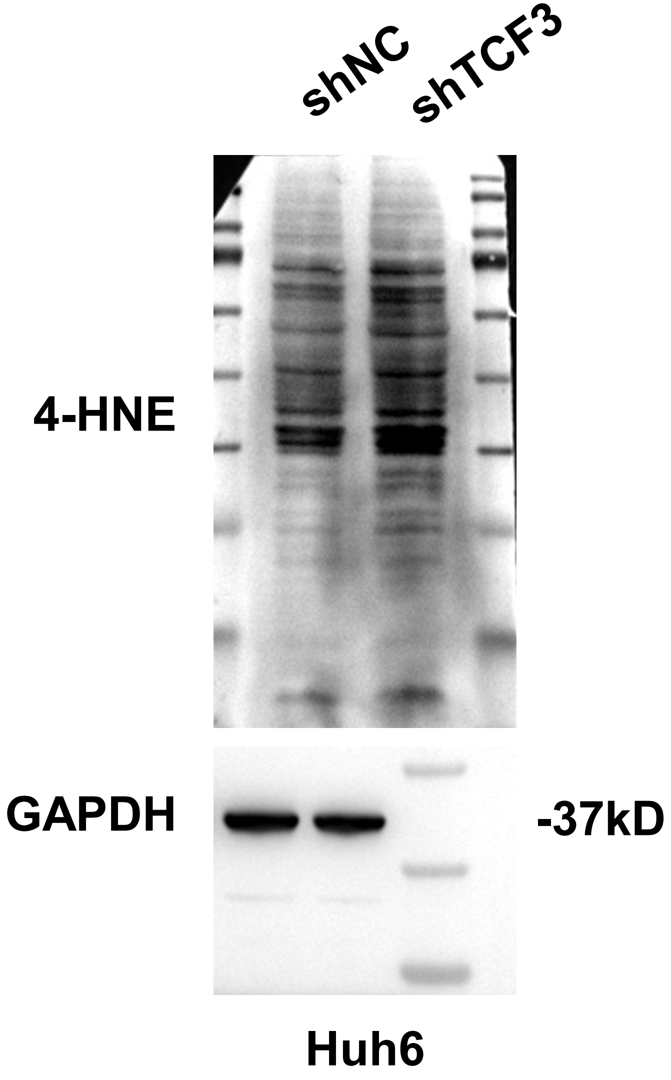

Supplement: Supplementary file 2 — Supplementary Material 2. [file 13046_2025_3587_MOESM2_ESM.pdf]
