# Supplementary material for: TCF3 activates super-enhancer-driven TRIB2 overexpression to suppress ferroptosis and promote hepatoblastoma proliferation
Source: J Exp Clin Cancer Res. 2025 Dec 29;44:329. doi: 10.1186/s13046-025-03587-1 (PMC12750601; doi:10.1186/s13046-025-03587-1)
Supplement: Supplementary file 3 — Supplementary Material 3. [file 13046_2025_3587_MOESM3_ESM.docx]

**Supplementary Figures and Legends**

**
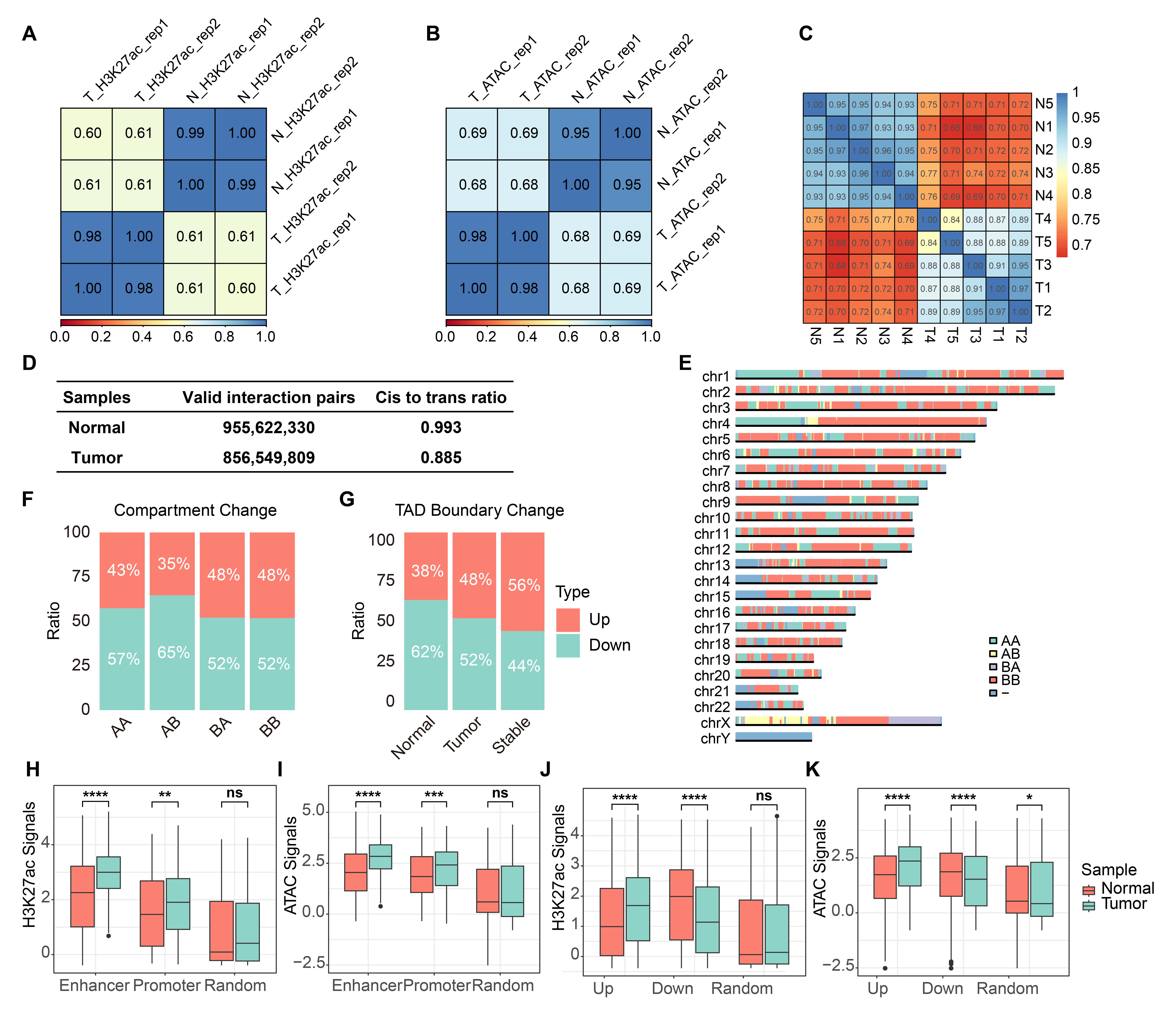
**

**Supplementary Figure 1: Validation of 3D genome alterations in HB**

(A-C) Correlation coefficients among replicates based on H3K27ac CUT&Tag (A), ATAC-seq (B) and RNA-seq (C). (D)​​ Valid interaction pairs and *cis*-trans ratios of Hi-C. (E) The switched compartment in the whole genome. (F) Proportion of DEGs associated with A/B compartment switching. (G) Proportion of DEGs flanking tissue-specific TAD boundaries. (H-I) H3K27ac CUT&Tag (H) and ATAC-seq (I) signal quantifications at HB-specific E-P loop anchors. (J-K) H3K27ac CUT&Tag (J) and ATAC-seq (K) signal quantifications at promoters of DEGs.


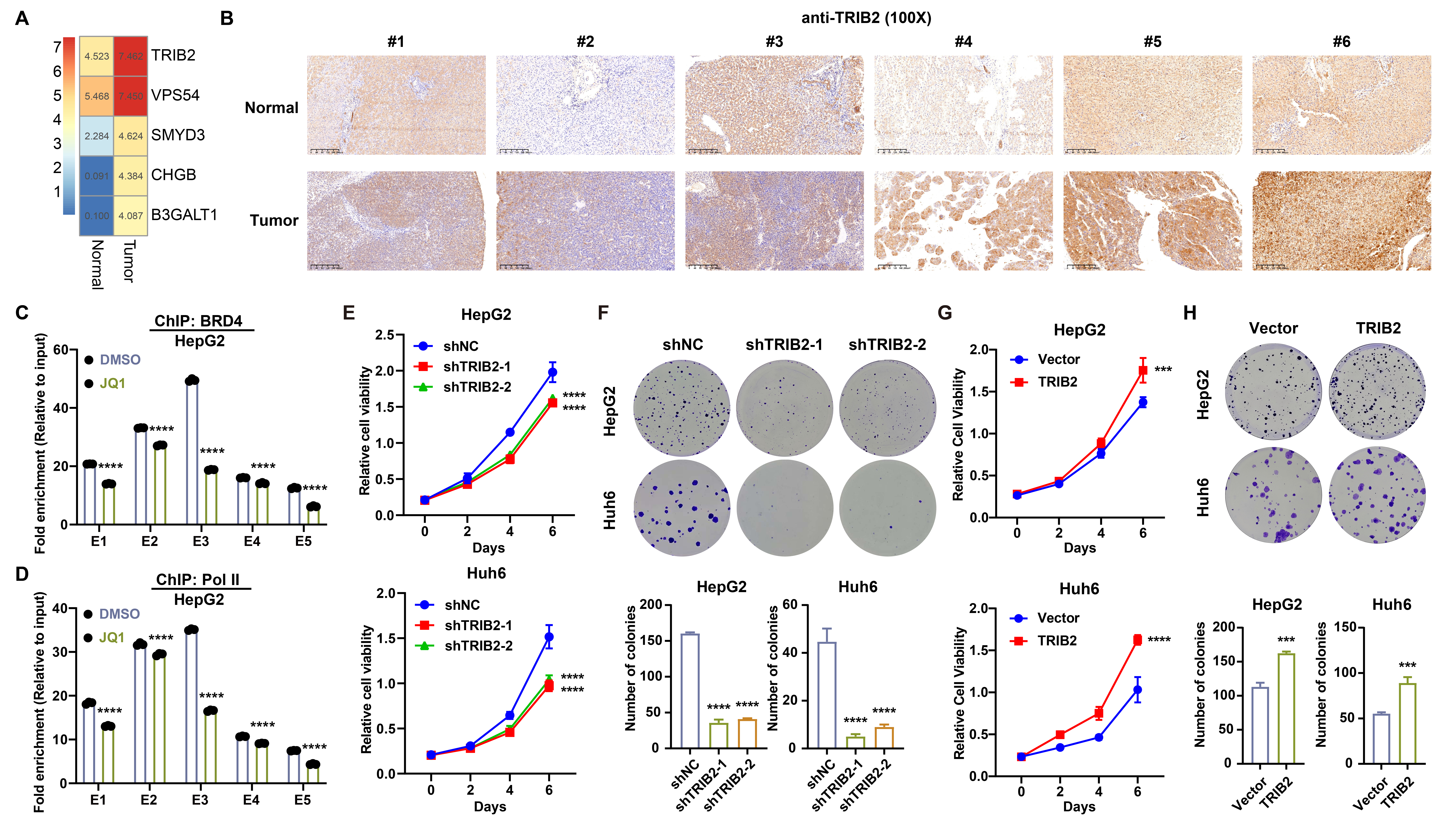


**Supplementary Figure 2:** **TRIB2 expression profile and functional impact on HB cell proliferation**

(A) Heatmap of gene expression (TRIB2, VPS54, SMYD3, CHGB, B3GALT1) in non-tumor and tumor tissues. (B) Validation of TRIB2 overexpression in HB clinical specimens via IHC staining (n = 6 paired samples). (C-D) ChIP-qPCR assays suggested the occupancy of BRD4 (C) and RNA Pol II (D) at TRIB2 enhancer elements with or without JQ1 treatment (10μM, 24h; n = 3 independent biological replicates). (E) CCK-8 assay in HB cells with or without TRIB2 knockdown at indicated time points (n = 5 independent biological replicates). (F) Colony formation assay and quantification in HB cells with or without TRIB2 knockdown (n = 3 independent biological replicates). (G) CCK-8 assay in HB cells with or without TRIB2 overexpression at indicated time points (n = 5 independent biological replicates). (H) Colony formation assay and quantification in HB cells with or without TRIB2 overexpression (n = 3 independent biological replicates).


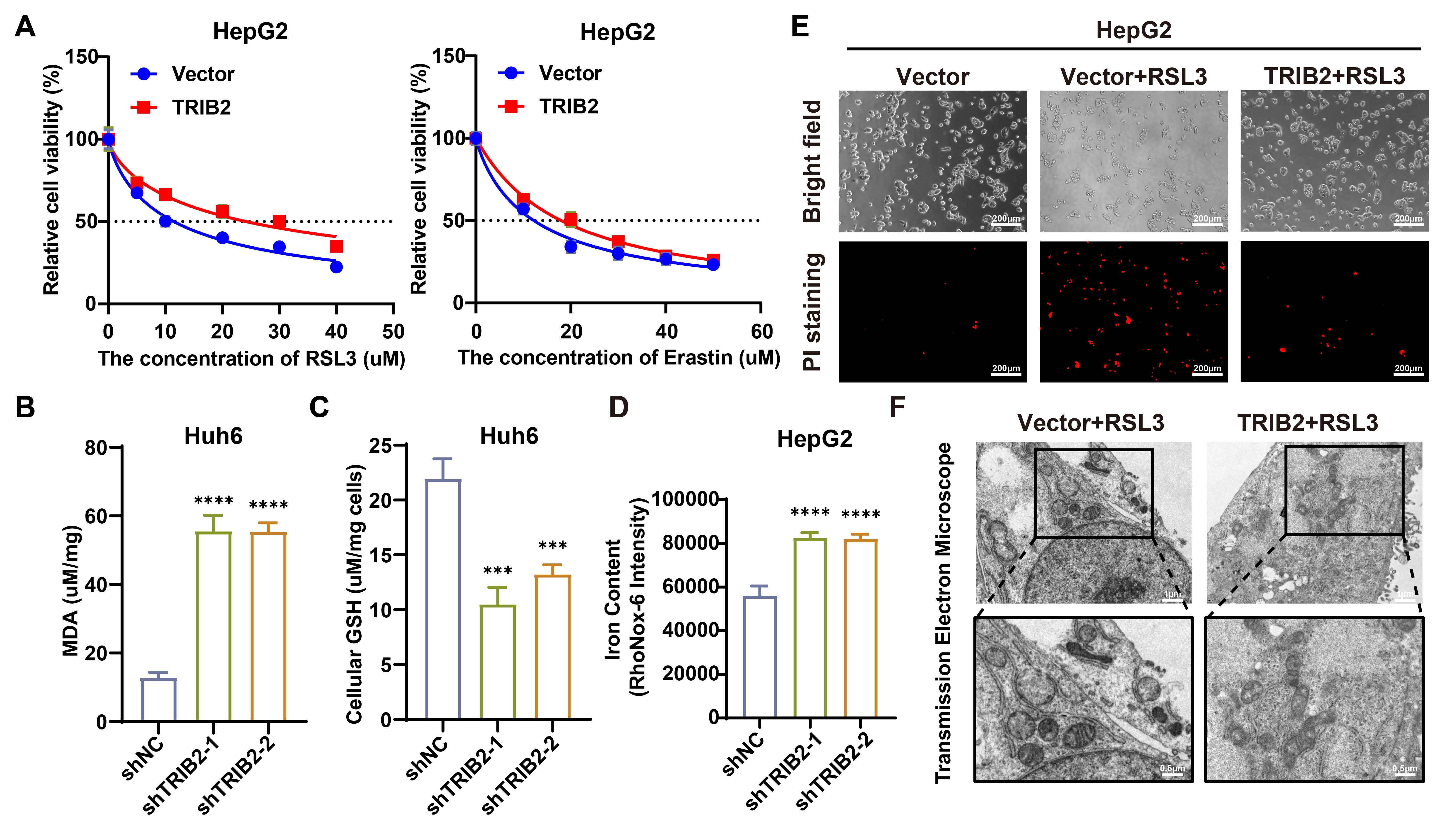


**Supplementary Figure 3: Validation of ferroptosis induction by TRIB2 depletion in HB cells**

(A) Dose-response curves for HB cells with or without TRIB2 overexpression treated with RSL3 or Erastin (n = 5 independent biological replicates). (B-C) MDA (B) or GSH (C) levels in Huh6 cell lysates with or without TRIB2 knockdown (n = 3 independent biological replicates). (D) Labile iron pool levels of HepG2 cells with or without TRIB2 knockdown (n = 5 independent biological replicates). (E) Cell death measured by PI staining of the HepG2 cells with or without TRIB2 overexpression treated with RSL3, scale bar, 200μm. (F) Transmission electron micrographs showing mitochondrial ultrastructure in HepG2 cells with or without TRIB2 overexpression treated with RSL3, scale bar, upper 1 µm, lower ‌500 nm.


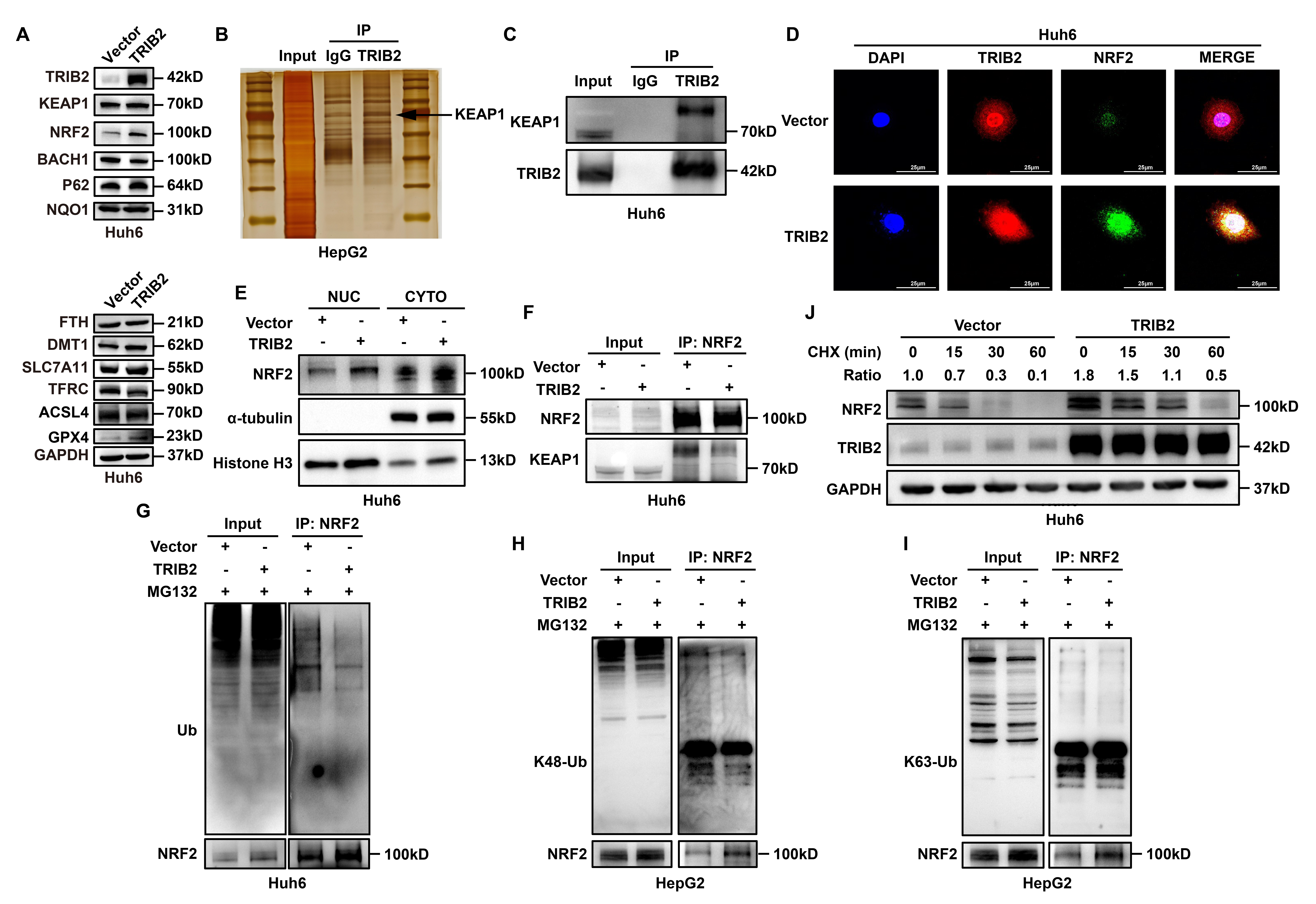


**Supplementary Figure 4: Validation of TRIB2-KEAP1 interactions and downstream effects on NRF2**

(A) Western blots of ferroptosis-related regulators in Huh6 cells with or without TRIB2 overexpression. (B) Silver staining of TRIB2 immunoprecipitates from HepG2 cells. (C) IP of TRIB2 to verify the interaction between TRIB2 and KEAP1 in Huh6 cells. (D) Immunofluorescence imaging of NRF2 in Huh6 cells with or without TRIB2 overexpression (cyan: NRF2; red: TRIB2; blue: DAPI). scale bar, 25 μm. (E) Western blots of the Huh6 cell lysates by nuclear-cytoplasmic fractionation with or without TRIB2 overexpression. Markers: Histone H3 (nucleus), α-tubulin (cytoplasm). (F) IP of NRF2 to verify the interaction between NRF2 and KEAP1 in Huh6 cells with or without TRIB2 overexpression. (G) IP of NRF2 to verify NRF2 ubiquitination under MG132 treatment (10 μM, 6h) in Huh6 cells with or without TRIB2 overexpression. (H-I) IP of NRF2 to verify K48-linked (H) and K63-linked (I) ubiquitination under MG132 treatment (10 μM, 6h) in HepG2 cells with or without TRIB2 overexpression. (J) Western blots of the decay rate of NRF2 protein detected at indicated time points under the treatment of CHX (100 μg/ml) in Huh6 cells with or without TRIB2 overexpression.


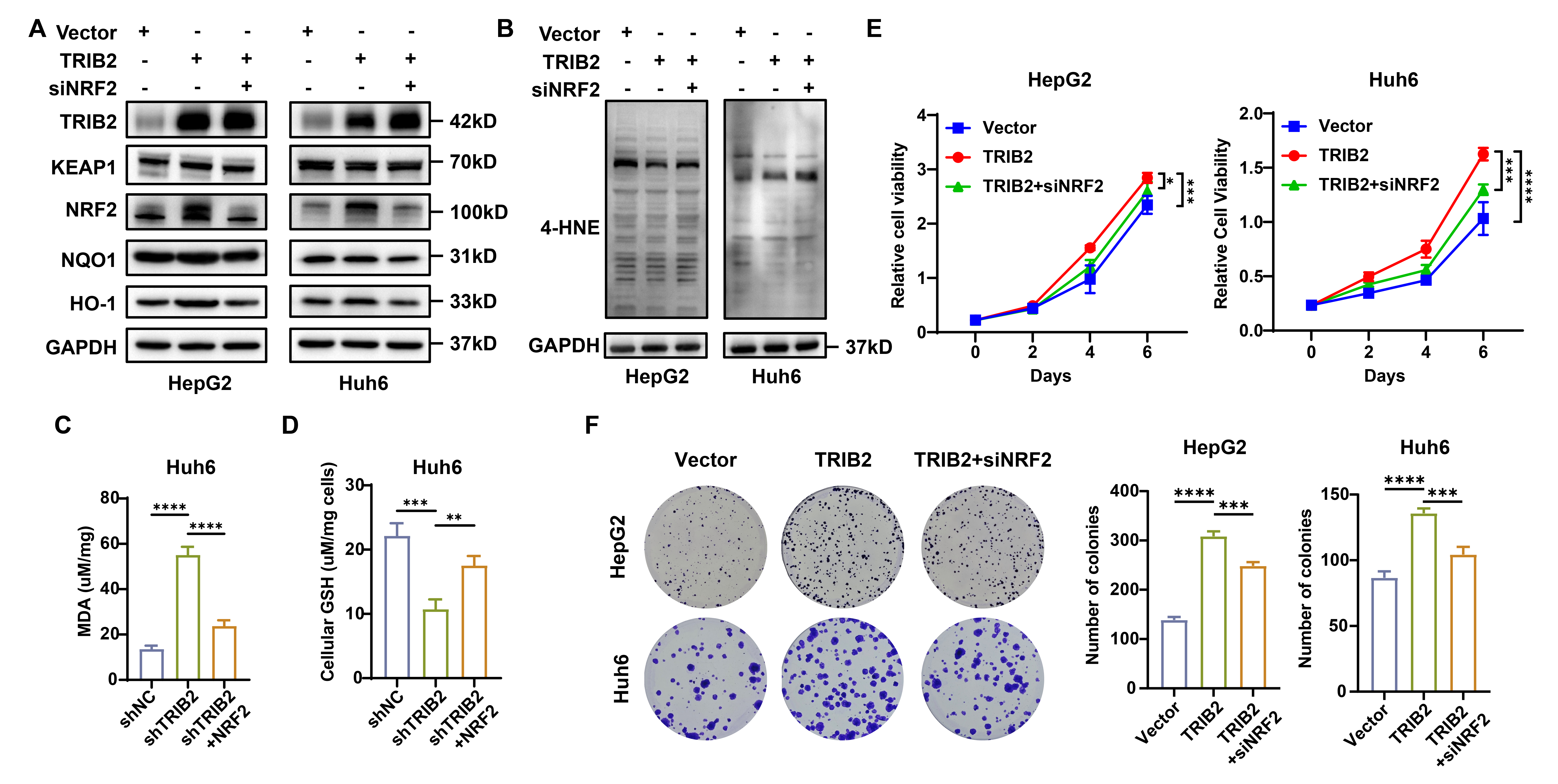


**Supplementary Figure 5:** **Validation of TRIB2-NRF2 axis in modulating ferroptosis sensitivity and antioxidant response**

(A-B) Western blots of NRF2, HO-1, NQO1 (A), and 4-HNE (B) in TRIB2-overexpression HB cells with or without NRF2 knockdown. (C-D) MDA (C) or GSH (D) levels in TRIB2-knockdown Huh6 cell lysates with or without NRF2 overexpression (n = 3 independent biological replicates). (E) CCK-8 assay in TRIB2-overexpression HB cells with or without NRF2 knockdown at indicated time points (n = 5 independent biological replicates). (F) Colony formation assay and quantification in TRIB2-overexpression HB cells with or without NRF2 knockdown (n = 3 independent biological replicates).


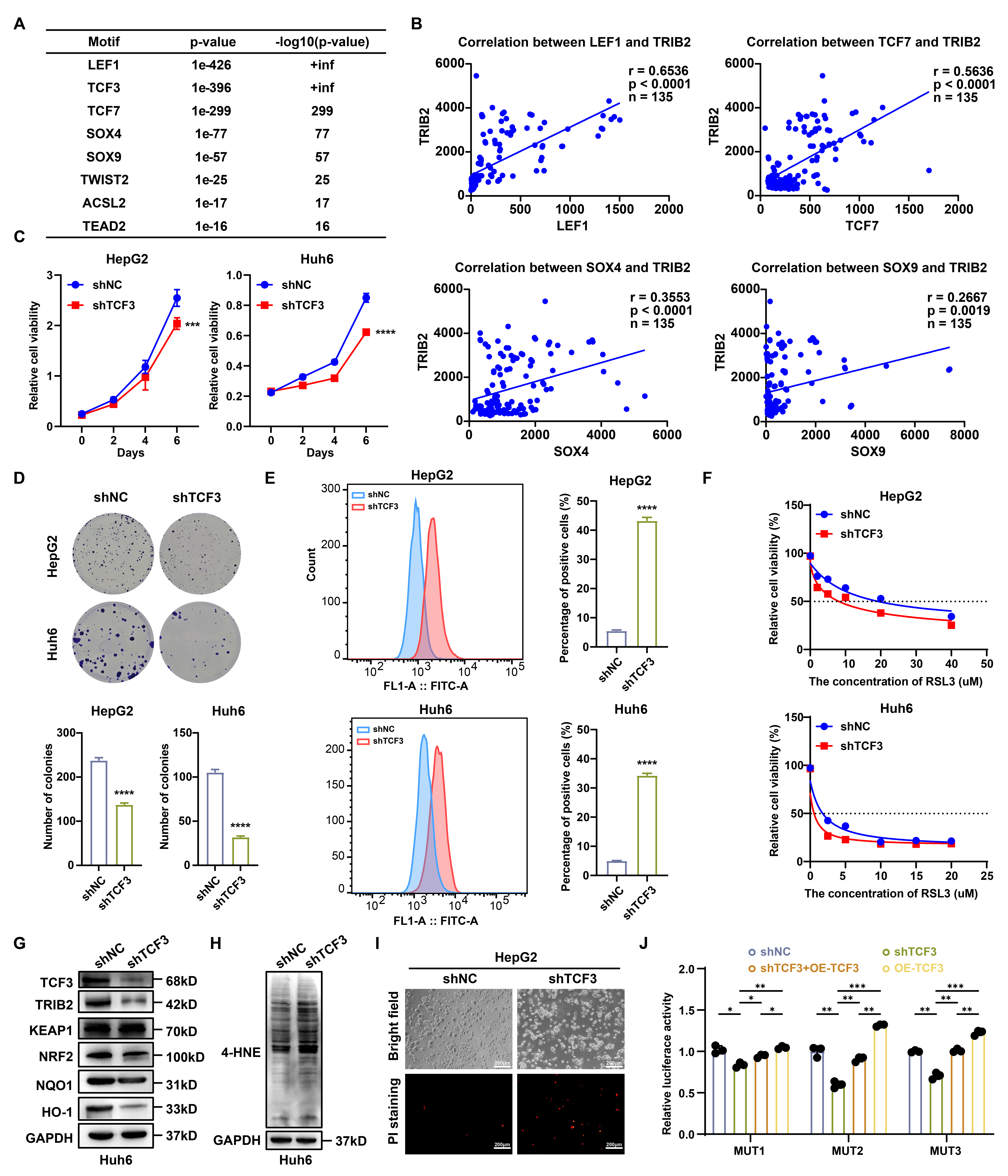


**Supplementary** **Figure 6: Validation of TCF3-mediated functional and ferroptosis regulation**

(A) Intersection of HB-specific enhancer motifs and up-regulated genes, showing significant p-values for related TFs. (B) Correlation analysis between TRIB2 and individual TFs (LEF1, TCF7, SOX4, SOX9) in HB datasets (GSE132219 and GSE104766). (C) CCK-8 assay in HB cells with or without TCF3 knockdown at indicated time points (n = 5 independent biological replicates). (D) Colony formation assay and quantification in HB cells with or without TCF3 knockdown (n = 3 independent biological replicates). (E) Flow cytometry quantification of lipid ROS using BODIPY-C11 probe oxidation in HB cells with or without TCF3 knockdown (n = 3 independent biological replicates). (F) ​​Dose-response curves for HB cells with or without TCF3 knockdown treated with RSL3 (n = 5 independent biological replicates). (G-H) Western blots of TRIB2, NRF2, HO-1, NQO1 (G), and 4-HNE (H) in Huh6 cells with or without TCF3 knockdown. (I) Cell death measured by PI staining of the HepG2 cells with or without TCF3 knockdown, scale bar, 200μm. (J) Luciferase reporter assays showing TRIB2 promoter activity regulated by TCF3 overexpression, knockdown, or binding site mutation (n = 3 independent biological replicates).

**
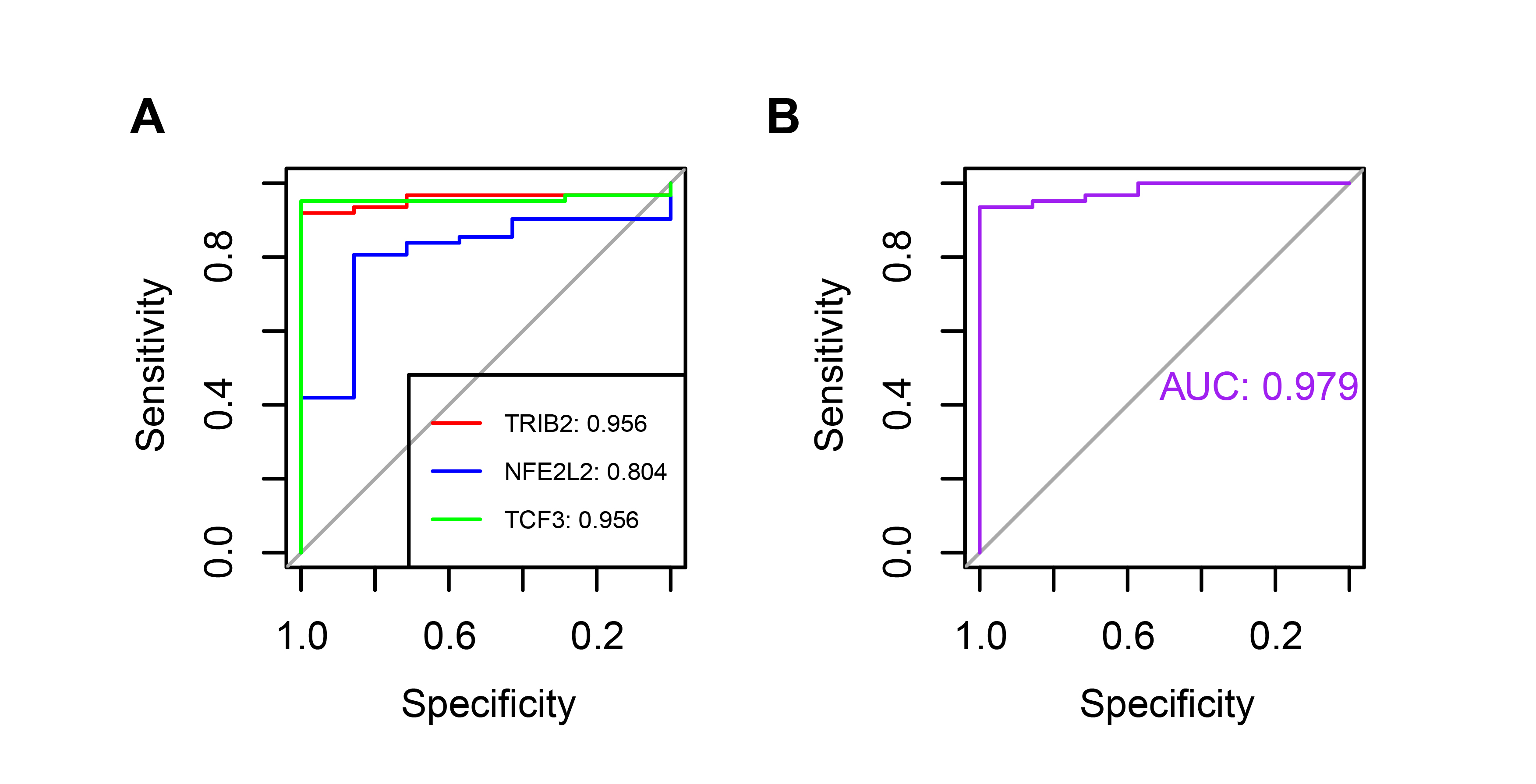
**

**Supplementary** **Figure 7: Diagnostic and prognostic validation of TCF3-TRIB2-NRF2 axis**

(A-B) Diagnostic ROC curves for individual markers (A) and TCF3-TRIB2-NFE2L2 combined signature (B) in HB cohorts (n = 35 HB and 7 non-tumor samples).
